# Prediction and Prioritisation of Novel Anthelmintic Candidates from Public Databases Using Deep Learning and Available Bioactivity Data Sets

**DOI:** 10.3390/ijms26073134

**Published:** 2025-03-28

**Authors:** Aya C. Taki, Louis Kapp, Ross S. Hall, Joseph J. Byrne, Brad E. Sleebs, Bill C. H. Chang, Robin B. Gasser, Andreas Hofmann

**Affiliations:** 1 Department of Veterinary Biosciences, Melbourne Veterinary School, Faculty of Science, The University of Melbourne, Parkville, VIC 3010, Australia; aya.taki@unimelb.edu.au (A.C.T.); kapplouis@gmail.com (L.K.); rossh@unimelb.edu.au (R.S.H.); byrnej1@unimelb.edu.au (J.J.B.); sleebs@wehi.edu.au (B.E.S.); billc@unimelb.edu.au (B.C.H.C.); 2 Institute of Cognitive Science, University of Osnabrück, 49090 Osnabrück, Germany; 3 Walter and Eliza Hall Institute of Medical Research, Parkville, VIC 3052, Australia; 4 Max Rubner-Institut, Federal Research Institute of Nutrition and Food, 95326 Kulmbach, Germany

**Keywords:** antiparasitics, artificial neural network, drug discovery, parasite, parasitic nematode

## Abstract

The control of socioeconomically important parasitic roundworms (nematodes) of animals has become challenging or ineffective due to problems associated with widespread resistance in these worms to most classes of chemotherapeutic drugs (anthelmintics) currently available. Thus, there is an urgent need to discover and develop novel compounds with unique mechanisms of action to underpin effective parasite control programmes. Here, we evaluated an in silico (computational) approach to accelerate the discovery of new anthelmintics against the parasitic nematode *Haemonchus contortus* (barber’s pole worm) as a model system. Using a supervised machine learning workflow, we trained and assessed a multi-layer perceptron classifier on a labelled dataset of 15,000 small-molecule compounds, for which extensive bioactivity data were previously obtained for *H. contortus* via high-throughput screening, as well as evidence-based datasets from the peer-reviewed literature. This model achieved 83% precision and 81% recall on the class of ‘active’ compounds during testing, despite a high imbalance in the training data, with only 1% of compounds carrying this label. The trained model was then used to infer nematocidal candidates by in silico screening of 14.2 million compounds from the ZINC15 database. An experimental assessment of 10 of these candidates showed significant inhibitory effects on the motility and development of *H. contortus* larvae and adults in vitro, with two compounds exhibiting high potency for further exploration as lead candidates. These findings indicate that the present machine learning-based approach could accelerate the in silico prediction and prioritisation of anthelmintic small molecules for subsequent in vitro and in vivo validations.

## 1. Introduction

Acute and chronic infectious diseases caused by viruses, bacteria, fungi, and parasites have major, adverse impacts on the health and welfare of animals and humans worldwide. Parasites have a pronounced impact because they often cause chronic infections or diseases and can be very challenging to control due to their complex biology, ability to evade host immune responses, and the lack of effective vaccines or treatments against many of them.

The global disease burden in humans imposed by parasitic worms (i.e., soil transmitted helminths, STHs) is presently estimated at ~2 million disability-adjusted life years [1,2,3], while the annual economic impact caused by helminth diseases and associated productivity losses in farm animals (cattle, sheep, goats, and pigs) is predicted to be at least tens of billions of dollars per annum [4,5,6].

Gastrointestinal roundworms (nematodes) of the order Strongylida (‘strongylids’) of livestock animals, such as species of *Haemonchus*, *Ostertagia*, *Trichostrongylus*, *Cooperia*, *Nematodirus*, *Oesophagostomum* and *Chabertia*, are recognised as some key parasites of livestock animals [7,8,9]. Many of them have become very challenging to control with chemotherapeutic drugs (anthelmintics) because populations of these parasites have become resistant to treatment due to their excessive use of these drugs on farms [10,11,12]. While vaccination would be a preferred approach to preventing infections in animals, no vaccines are commercially available for these parasites [13], with the exception of Barbervax^®^ against *H. contortus* (see [14]); however, the protection induced by this vaccine is variable and not long-lasting [13,15]. Thus, given the widespread resistance problem in strongylid nematodes of livestock to all/most current classes drugs (e.g., [16,17,18,19,20]), there is a major demand for novel anthelmintics [11,21,22].

*Haemonchus contortus* is a key representative for related nematode species in the order Strongylida, and has become a unique model system for applied and fundamental research because of the availability of established tools (e.g., drug screening platforms) and resources (including laboratory strains of the parasite, multiple ‘omic—datasets and, importantly, extensive high-throughput screening datasets) [23,24,25,26,27,28,29,30,31,32,33]. Over the past 15 years, in public–private partnerships, we have been focussing on the discovery of novel anthelmintic entities by combining high-throughput screening, structure activity relationship (SAR) studies, and medicinal chemistry optimisation (e.g., [34,35,36,37]). During this time, we have been able to establish a large set of bioactivity data for *H. contortus* for small molecules. To accelerate the discovery of new anthelmintic chemotypes, we assess here the potential to harness these in-house bioactivity datasets augmented with published, evidence-based data to develop an in silico model for the prediction and prioritisation of novel small-molecular anthelmintic candidates represented in public chemical databases.

Computational approaches, particularly quantitative structure–activity relationships (QSAR) modelling, have had a profound impact on drug discovery [38]. Methodologically, QSAR modelling involves the conversion of a set of chemicals with experimentally determined biological properties into molecular descriptors (physico-chemical parameters and/or fingerprints). Using statistical methods, relationships between descriptors and the biological properties of interest can be established [39,40]. Whereas early QSAR applications employed linear regression models [41,42,43], these were subsequently replaced by Bayesian neural networks [44,45], followed by Random Forests [46,47] and support vector machines [48]. Statistically validated QSAR models represent a helpful tool for the in silico screening of new chemicals with the desired biological properties. The availability of large bioactivity datasets for chemical compounds [49,50], the development/definition of thousands of molecular descriptors, and increased popularity of in silico approaches resulted in the widespread use of QSAR for a diverse array of biological properties relevant to drug discovery [51,52,53]. However, modelling biological properties of big datasets has posed a challenge for classical machine learning algorithms [54,55], leading to the application of deep learning methods (artificial neural networks) as a practical solution [56,57]. Deep learning is particularly well suited for QSAR modelling because it possesses multiple hidden layers capable of computing adaptive non-linear features that increasingly capture complex data patterns with each iterative additional layer, which makes this approach useful for tackling more complex chemical data [58,59].

**Figure 1:**
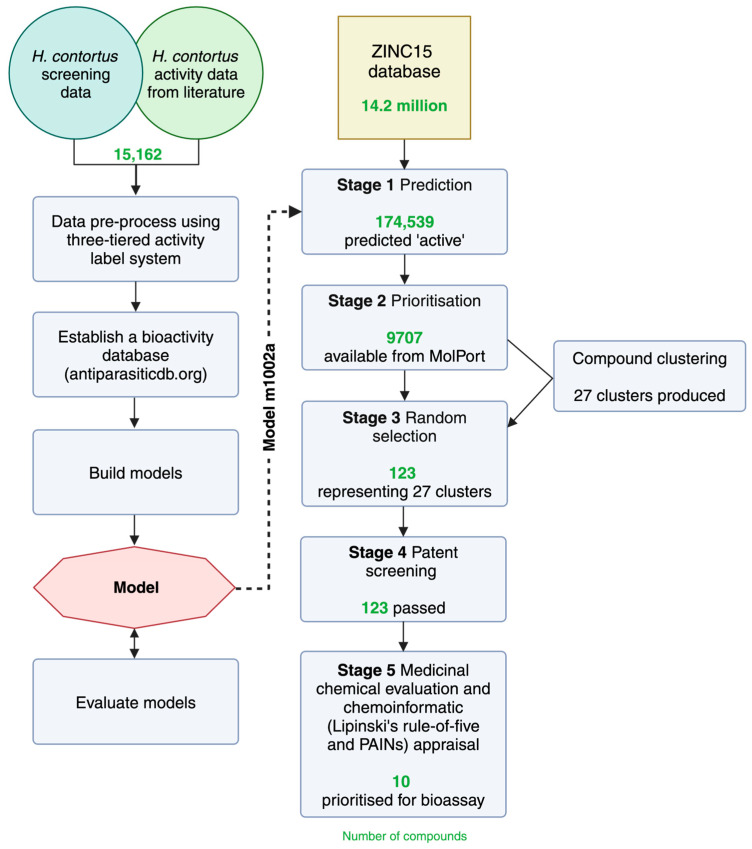
An overview of the approach taken in the prediction and prioritisation of anthelmintic candidates from a public database. Bioactivity data collected from an in-house screening dataset and available from the literature against *Haemonchus contortus* were utilised, encompassing information on a total of 15,162 compounds. These data were employed to train, build, and evaluate machine learning models for predicting active molecules against *H. contortus* (left side tree). The derived model, m1002a, was used to predict ‘active’ compounds within the ZINC15 database, which consists of 14.2 million small molecules. Subsequently, the predicted ‘active’ compounds underwent a rigorous prioritisation and evaluation process, resulting in the selection of 10 compounds for bioassay validation (right side tree).

Here, we introduce a classification model based on a multi-layer perceptron that was trained using our in-house bioactivity (motility) datasets, as well as evidence-based data published in the peer-reviewed literature. This model was used to screen 14.2 million compounds within the public database, called ZINC [60]. From a subset of small molecules in the ZINC15 database predicted to be nematocidal in silico, 10 representative structurally distinct compounds were selected and subjected to experimental evaluation in vitro. A schematic overview of our approach is provided in Figure 1.

## 2. Results

### 2.1. Modelling Approach

Initial attempts to establish a regression model for the in-house *H. contortus* motility data for 14,864 small-molecule compounds from the *Open Scaffolds Collection* and *Pathogen Box* [26,61] did not yield models with acceptable performances with machine learning (support vector machine—scikit-learn) (Table S1) or deep learning (neural network—Keras/TensorFlow MLP) (Table S2). As an alternative to the regression approach, we turned to testing neural network-based classification models, aimed at the prediction of categorical labels.

### 2.2. Data Curation and Establishment of Publicly Accessible Database

Taking advantage of the normalisation effect concurrent with categorising numerical data, bioactivity data of small-molecule compounds from phenotypic assays other than the whole-organism assay established in-house [62] were also considered. A three-tier labelling system that classified ‘active’, ‘weakly active’ and inactive (label: ‘none’) compounds was devised and rules for mapping numerical data from different phenotypic assays (i.e., Wiggle index, viability, reduction, EC_50_, and MIC_75_) were defined (Table 1). Applying these classification rules to bioactivity data reported in 21 publications, including the data obtained from the screening of the *Open Scaffolds Collection* and *Pathogen Box*, a bioactivity dataset consisting of 15,162 small-molecule compounds was assembled (Table 2).

While data preparation and labelling according to the chosen classification rules was a straightforward procedure for in-house data, this process was labour-intensive and time-consuming for data from peer-reviewed literature, due to the way in which they are presented and organised in conventional scientific articles. Much of the relevant information in medicinal chemistry articles is embedded in the text or in tables and requires a re-establishment of the chemical identity of small molecules by a chemist.

Making these efforts worthwhile beyond the present study, we decided to assemble the labelled data extracted from the peer-reviewed literature into an online database (https://antiparasiticsdb.org/, accessed 26 March 2025) and make this resource available to the scientific community. As of May 2024, the database contains information on 890 small-molecule compounds and four peptides with respect to their bioactivity (classified using the criteria summarised in Table 1); evidence of resistance to one or more of these compounds in one or more of 104 parasitic and free-living organisms; and assay methodologies and other quantitative data (where available)—extracted from 83 peer-reviewed scientific articles, each linked to a digital object identifier (DOI). The small-molecule compound information includes name and compound class, the SMILES string, the CAS registry number and links to the PubChem (https://pubchem.ncbi.nlm.nih.gov/; accessed on 26 March 2025) and ChEMBL databases (https://www.ebi.ac.uk/chembl/; accessed on 26 March 2025) where available. For peptides, links to the NCBI Protein database (https://www.ncbi.nlm.nih.gov/protein/; accessed on 26 March 2025) and the Protein Data Bank (https://www.rcsb.org/, accessed on 26 March 2025) are provided where available.

### 2.3. Feature Generation, Model Architecture, and Training

Based on experiences from preliminary modelling attempts, in which different small-molecule representations were tested, we decided to proceed with Morgan extended connectivity fingerprints (8192 bits), augmented with 200 calculated physico-chemical descriptors (1600 bits). The resultant feature vector obtained from SMILES representations of small-molecule compounds thus consisted of 9792 bits.

For the testing of model architectures, the dataset of 15,162 compounds (Table 2) was split into a global training (80%) and a global test dataset (20%); refer to Table 3 for details. The global training dataset was used for training models with varying architectures in a grid search and three-fold cross-validation. For each cross-validation run, the global training dataset was split further into training (75%) and validation (25%) datasets.

**Figure 2:**
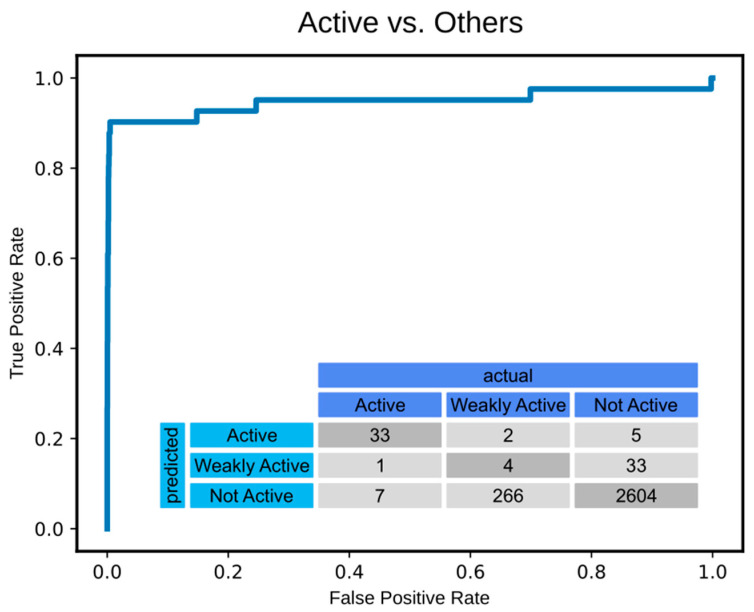
The fit of the logistic regression model m1002a with the test data. The fit of model m1002a with the test data is illustrated by the ROC curve for distinguishing ‘active’ compounds from those labelled as ‘weakly active’ and inactive (‘none’). The inset shows the confusion matrix.

Grid searches were carried out in four different series (1001–1004) to test different numbers of hidden layers (*n_hidden* = 1, 2) as well as different dimensions of the hidden layers using the cross-entropy loss function. A fifth series (2001) was run to screen the same parameters as for series 1001 but using the Macro F1 Score loss function. From these grid searches, a model architecture from series_1002, consisting of two hidden layers with dimensions of *dim_hidden_1* = 75 and *dim_hidden_2* = 5, was identified as the one delivering the best recall for the ‘active’ label. The model architectures and performances of the five series are summarised in Table 4, and the results for the model architectures tested are given in Tables S3–S7. Using the best performing model architectures of each series, classification predictions were made for the global test dataset, and the comparison of precision and recall statistics confirmed the identified model architecture as the best performing one. The model with the chosen architecture and the weights obtained in the first of the three cross-validation runs was saved for further usage (model m1002a). The statistics for the fit of model m1002a with the training data are shown in Figure 2.

### 2.4. In Silico Screening and Post-Processing

A subset of the ZINC15 database comprising 14.2 million compounds [60] were encoded from their SMILES representations to yield feature vectors. The encoded compounds were subjected to classification prediction of nematocidal activity against *H. contortus* using model m1002a. In total, 174,539 molecules (1.2%) were predicted as ‘active’ compounds (Table 5), of which 9707 were available for purchase through the vendor MolPort.

### 2.5. Clustering of Compounds with Predicted Nematocidal Activity

**Figure 3:**
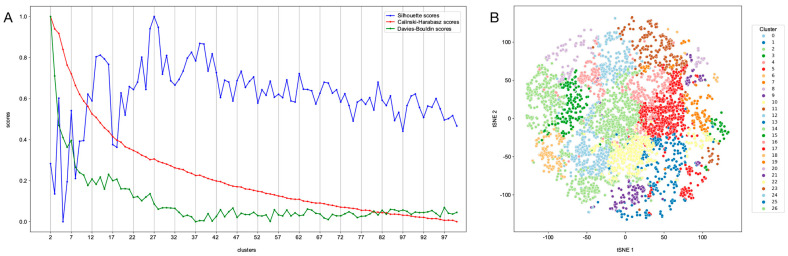
Compound clustering. (**A**) A plot of the three evaluation metric scores (*y*-axis) vs. number of clusters (*x*-axis). A more meaningful set of clusters is characterised by (i) a higher Silhouette Score (blue), (ii) a higher Calinski–Harabasz Index (red), and (iii) a lower Davies–Bouldin Index (green). Based on the results shown, the optimum number of clusters was determined as *k* = 27. (**B**) Visualisation of the 27 compound clusters using *t*-SNE to reduce the embedding dimensionality from 32 to 2.

To guide the selection of compounds for experimental evaluation, the set of 174,539 molecules from the ZIN15 database predicted to be ‘active’ was analysed using a clustering methodology that combines PCA and a Variational Autoencoder to consider the global and local molecular features in an integrated manner [81]. Using the *k*-means algorithm, different clustering calculations were made with *k* varying between 2 and 100. Based on analysis of the Silhouette, Calinski–Harabasz, and Davies–Bouldin scores, the number of *k* = 27 clusters was inferred to be optimal for this particular application (Figure 3). Therefore, the set of 17,539 compounds was subdivided into 27 clusters, each of which contained compounds with similar molecular frameworks, effectively representing a mix of diverse structures for subsequent selection.

### 2.6. Selection of Molecules for Experimental Evaluation

From all 9707 compounds readily available via MolPort, up to five were randomly selected from each of the 27 clusters as potential candidates for experimental testing. This resulted in 123 compounds (see Table S8), since four clusters contained less than five compounds. Subsequently, these 123 compounds were screened for potentially existing patent claims via automated searches of the web resource Google Patents and evaluated chemoinformatically for drug-likeness and an absence of known PAINs motifs. None of these were linked to a patent or any of the filtering keywords, such that no compound was excluded at this step. In a last filtering step, the list was scrutinised by a highly experienced medicinal chemist, also considering the results from the chemoinformatic analyses, resulting in the selection of 10 compounds with favourable drug-like structures, molecular weights within the range of 200–500 Da, and no PAINS motifs (Table 6 and Table S9) for subsequent experimental validation. Some of the 10 compounds selected were within the same cluster (i.e., compounds **1** and **3** were in cluster 10; **2** and **8** were in cluster 25; and **6** and **10** were in cluster 7). In addition, 20 compounds from the ZINC15 database predicted as inactive (‘none’) against *H. contortus* were randomly selected as negative controls (Table S10) for the experimental assessment.

The prioritisation strategy used (clustering, filtering by patent status and medicinal chemistry appraisal) reflects a practical pipeline, commonly applied for discovery, to ensure structural diversity, novelty, commercial availability and suitability for further lead optimisation. Although this study was conducted in an academic setting, these considerations were deemed essential to identify promising and developable leads for subsequent translational efforts.

### 2.7. Experimental Evaluation of Molecules with Predicted Bioactivity Against H. contortus

The activity of the 10 compounds prioritised as ‘active’ and the other 20 compounds predicted to be not active (‘none’) against exsheathed third-stage larvae (xL3) of *H. contortus* were individually tested in a dose–response manner, with potency expressed as half-maximal inhibitory concentration (IC_50_) and maximum motility inhibition (MMI) (Table 6). After 168 h, all 10 ‘active’ compounds exhibited inhibitory effects on larval motility (Table 6), resulting in 42–100% MMIs at 50 µM (Figure S1) and none of the other 20 compounds affected the motility of xL3s (Figure S2). Moreover, 9 of the 10 compounds achieved IC_50_ values ranging from 0.8 µM to 7.9 µM (excluding compound **5**, with IC_50_ 37.5 ± 2.5 µM). Notably, compounds **4** and **6** inhibited motility most, resulting in MMIs of 90% (IC_50_ = 5.8 µM) and 100% (IC_50_ = 7.4 ± 9.4 µM), respectively. Compounds **1**, **2**, **3** and **7** also showed moderate inhibitory effects, with MMIs in the range of 50–70% (50%, 53%, 54% and 70%, respectively), coupled with IC_50_ values of <10 µM (6.2 ± 0.3 µM, 1.8 ± 1.4 µM, 7.9 ± 1.9 µM and 2.1 ± 3.5 µM, respectively). Compounds **8** and **10** exhibited the lowest IC_50_ values (0.8 ± 1.6 µM and 0.8 ± 1.2 µM), comparable to that of moxidectin (MMI of 5%; IC_50_ = 0.5 µM), but only attaining MMIs of 42% and 50%.

In addition to assessing xL3 motility reduction, the 10 predicted ‘active’ + 20 predicted inactive (‘none’) compounds were evaluated for adverse effects on larval development and/or worm morphology (Table 6). Compounds **3** and **6** inhibited larval development by 100% and induced abnormal phenotypes—both compounds induced an *evisceration* (*Evi*) phenotype in 80% (compound **3**) and 100% (compound **6**) of larvae at 50 µM (Table 6). Compound **1** induced *curved* (*Cur*) and *Evi* phenotypes in 20% of larvae at 50 µM, although larval development was not inhibited (Table 6). As expected, the control compound, monepantel, induced a *coiled* (*Coi*) phenotype in 100% of larvae at 50 µM. No other compounds, including the other control compound, moxidectin, and the 20 inactive (‘none’) compounds, inhibited larval development or altered worm morphology.

**Figure 4:**
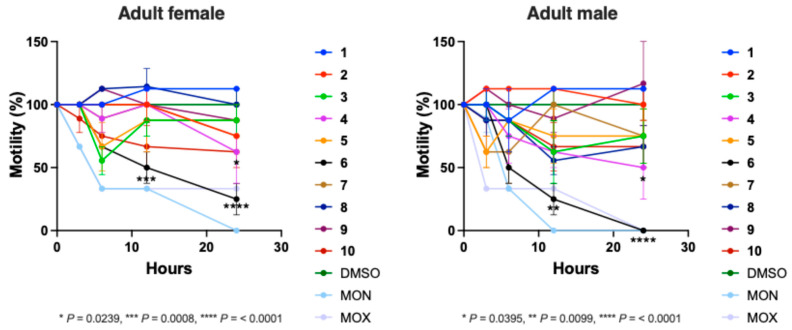
The bioactivity of ten prioritised small compounds against *Haemonchus contortus* adults. The inhibitory effect of compounds against adult females and males of *H. contortus* were assessed over a period of 24 h. Motility scores (assessed at 3 h, 6 h, 12 h and 24 h time points; cf. [37]) for each compound at 50 µM were calculated, normalised with reference to the negative control (DMSO; 100% motility), and recorded as a percentage. Two control compounds, monepantel (MON) and moxidectin (MOX), were included as references. Data points represent one experiment conducted in triplicate; the mean ± standard deviation (SD). A two-way ANOVA with Dunnett’s multiple comparison test was used to establish statistically significant differences against the DMSO control in worm motility for each time point. For compound names and identifiers, see Table S9.

Subsequently, the effect of the 10 predicted ‘active’ compounds to reduce the motility of adult females and males of *H. contortus* was assessed in a time-course assay (Figure 4). Compound **6** exhibited a significant inhibitory effect on both adult female and male worms, leading to a motility reduction of 33%, 50% and 75% (females) and 50%, 75% and 100% (males) over the time intervals (6 h, 12 h and 24 h, respectively). Notably, this effect was comparable to that of monepantel, which displayed respective motility reductions of 67%, 67% and 67% (females) and 67%, 67% and 100% (males) at the observed time points (6 h, 12 h and 24 h). Compounds **4** and **10** also significantly reduced the motility of adult worms by 40% (females; both compounds) and 50% (males; compound **4**) after 24 h.

The three most potent compounds (i.e., **3**, **4** and **6**) were evaluated for their cytotoxicity using HepG2 cells (human hepatoma cells) in vitro; no cytotoxic effects were observed for any of the three compounds over 48 h (CC_50_ values of >40 µM).

## 3. Discussion

### 3.1. Harnessing Existing In-House and Literature Data to Apply Machine Learning Methodologies

In recent years, the discovery of anthelmintics by the pharmaceutical industry has been slow compared with progress made in many medical areas focused on drugs against cancer, tuberculosis, and SARS-CoV-2. The most recent successes in commercially developed anthelmintics include emodepside [82], tribendimidine [83], monepantel [84], and derquantel [85], dating back to the 2000s. Currently, the discovery, characterisation, and evaluation of novel anthelminthic candidates still seems to be driven by academic research efforts, backed by tailored resources [86,87] and, to some extent, public–private partnerships [88,89,90].

The history of academic research in this area has afforded assays for the screening of small-molecule compounds for phenotypic changes in parasites in culture and, to a lesser extent, against validated/potential parasite molecular targets [91,92,93]. Whereas the amount of data accumulated in this area is rather small compared with the (proprietary) assay data available in large pharmaceutical companies, it still constitutes a valuable resource and, owing to the recent application of high-throughput technology in academic settings, is now reaching an extent that allows the application of deep learning approaches to this discovery process.

However, the direct application of such methodologies to the collective data resource in the scientific literature has been hampered by the fact that numerous different (semi-) quantitative metrics have been used to characterise the bioactivity of small-molecule compounds tested. Additionally, often, more than one developmental stage of a parasite of interest has been subjected to bioactivity screening assays, thus preventing a direct comparison of numerical outputs.

To address the challenge of directly comparing published bioactivity data of small-molecule compounds against *H. contortus*, in this study, we compiled relevant data from the scientific literature to use these in combination with previous data generated in-house. When employing machine learning to use the compiled data for training models, we normalised the disparate numerical data from different types of assays by transformation into classification labels and defined a set of criteria for these transformations (Table 1). Although alternative classifiers, such as Random Forests, were considered early in the model development process, they did not outperform the selected neural network model and were, thus, not pursued further once the deep learning approach yielded satisfactory recall and precision on the ‘active’ class. A similar approach based on two ‘rule books’ was used recently to standardise numerical bioactivity data from anti-schistosomiasis drug discovery to obtain a ‘severity score’ used for training with Bayesian machine learning methods [94]. Other attempts to leverage machine learning for drug discovery against schistosomiasis follow the same principle, albeit using less sophisticated classification rules and smaller sets of curated data [95].

We acknowledge the importance of model interpretability, particularly in the identification of features associated with biological activity. In our approach, feature importance was not directly assessed due to the nature of the composite input vector (Morgan fingerprints and calculated physico-chemical descriptors). However, in the future, we aim to complement this deep learning-based model with one or more simpler, interpretable models (e.g., Random Forests) trained on the same dataset, to extract descriptor importance and gain mechanistic insights into key molecular features associated with activity.

### 3.2. Prediction and Prioritisation of Novel Compounds with Nematocidal Properties and Assessment of Potential Leads

The experimental validation of the 10 prioritised compounds, computationally predicted to be bioactive, revealed that all compounds exhibited activity on *H. contortus* in vitro. All 10 assessed compounds exhibited inhibitory effects on larval motility, and two inhibited larval development and induced an abnormal phenotype. Furthermore, one compound, in addition to its pronounced effects on larvae, exhibited potent in vitro activity on adult worms of *H. contortus*.

Compounds **6** and **3** emerged as the most promising candidates among the compounds evaluated, displaying overall inhibition of both larval motility and development with IC_50_ values being only ~1 order of magnitude higher than those of monepantel. Notably, compound **6**—4-[(3-fluorophenyl)methoxy]benzonitrile—achieved 100% efficacy across all measured bioactivities for larvae (motility; development; abnormal phenotype). Similarly, compound **3**—6-(3,4-dimethoxyphenyl)pyrazine-2-carbonitrile—while reaching only 54% maximal motility inhibition, demonstrated 100% inhibition of larval development and induced an abnormal phenotype in treated larvae, proving its robust potency against *H. contortus*. Structurally (see also Table S11), compound **6** shares the substituted phenylether pharmacophore with two monepantel derivatives that possess antiparasitic activity, APDB C823 (CHEMBL402864, PubChem CID 11224875) and APDB C836 [68]. The methoxyphenyl-pyrazine core of compound **3** is a shared structural element with tetrahydroqunoxalines APDB C798, APDB C799 and APDB C802 for which antiparasitic activity was observed [69]. Both compounds induced an ‘*evisceration*’ phenotype in affected larvae, characterised by the protrusion of viscera (alimentary tract and surrounding tissues) through the excretory pore in xL3s [69], ultimately resulting in a lethal outcome for the larvae. Moreover, compound **6** achieved complete immobilisation of adult males as well as a significant reduction in the motility of adult females within 24 h, comparable to the effectiveness of established commercial anthelmintics, suggesting its potential to effectively stop the release of thousands (~4000) of eggs per day [96,97] and disrupting the worm life cycle.

These observations add to the significance of compounds **6** and **3** as potential candidates for further exploration in combating parasitic nematodes. Compound **6** has undergone relatively extensive investigation as a potential treatment for non-communicable diseases, specifically epilepsy and Parkinson’s disease, however, not as anti-infectives. Synthesised as an intermediate analogue, the unique conformation of this compound and its derivatives have been studied for anticonvulsant and neuroprotective activities. These investigations stemmed from the well-characterised capability of compounds featuring a biaryl-linked motif and a ((3-fluorobenzyl)oxy)phenyl pharmacophore, both presented within compound **6**, to modulate sodium channels for the reduction in neuronal hyperexcitability (for epilepsy), and to inhibit an oxidase (monoamine oxidase B) in the brain (for Parkinson’s disease) [98,99,100,101]. Furthermore, compounds exhibiting these features (i.e., safinamide) are also reported to modulate calcium channels in addition to sodium, consequently leading to the indirect alteration in the levels of glutamate and/or gamma-aminobutyric acid (GABA) [102]. These properties align with the established knowledge that many current anthelmintic drugs target neurotransmitters or ion channels, such as macrocyclic lactones (e.g., ivermectin, moxidectin) and nicotinic acetylcholine receptor agonists (e.g., levamisole, pyrantel) [103,104], thereby modulating the neuromuscular function of parasitic nematodes to induce paralysis in the worms. The observed neuromodulatory effects of this pharmacophore, aligned with established mechanisms in anthelmintic drugs, suggest a plausible target(s) or pathway for the nematostatic and nematocidal effects in *H. contortus* witnessed in the present study. This finding further highlights the multifaceted potential of compound **6** in therapeutic interventions and warrants further exploration as a lead compound.

In contrast, compound **3** is an under-explored molecule as an intervention, with limited investigations into its biological activity, despite the potency recorded in this study. Existing research has primarily positioned compound **3** as an intermediate molecule in the synthesis of therapeutic kinase inhibitors, targeting human DRAK2 (death-associated protein-related apoptotic kinase-2), as potential treatments for diabetes by inhibiting apoptosis of islet beta-cells (WO2023083330). Kinases of the same family (DAPK; death-associated protein kinase) were identified in *H. contortus* [105]. Thus, it is plausible that compound **3** might share a similar binding mode in *H. contortus*, although insights into the binding region must be sought using in silico docking methods [106]. As evidenced by this compound’s adverse and potentially lethal effects on *H. contortus*, coupled with a lack of comprehensive studies, there arises a compelling prospect that compound **3** may represent a novel chemical space with significant promise as an anthelmintic agent.

## 4. Materials and Methods

### 4.1. Small-Molecule Bioactivity Data

A dataset of small-molecule compounds and their bioactivity against *H. contortus* was assembled using results from the screening of 14,864 compounds from two libraries (*Open Scaffolds Collection* and *Pathogen Box*) [26,61]; bioactivity was measured/defined as a significant reduction in motility of xL3 of *H. contortus* (see [62]). Additionally, select evidence-based data from the literature were used. To allow the inclusion of data from disparate sources using distinct experimental methods, rule sets for the conversion of numerical assay data into a three-tiered activity label system, consisting of ‘active’, ‘weakly active’ and inactive (label: ‘none’), were established (see Table 1). The data assembled from the literature, in this context, were compiled as a database and are available at https://antiparasiticsdb.org/ (accessed on 26 March 2025); compound entries in this database are referenced in the manuscript as APDB. The resultant, combined dataset consisted of 15,162 compounds that were tested against *H. contortus* (Table 2). Our dataset exhibits a significant imbalance—a common challenge for the analysis of bioactivity data. Specifically, 1.1% of compounds were labelled as ‘active’, 8.9% as ‘weakly active’ and 90% as inactive (‘none’) (Table 3). This imbalance mirrors the inherent challenges in identifying compounds with potent biological effects (reviewed by [68]), as most tested compounds show minimal to no activity against the target organism.

### 4.2. Model

**Figure 5:**
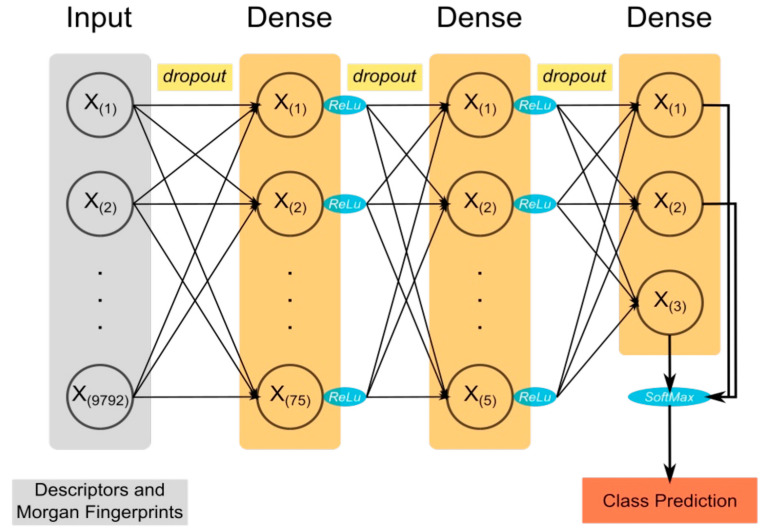
Model architecture. A schematic drawing of the model architecture and components. A dropout mechanism is applied between every fully connected layer to prevent over-fitting, and the rectified-linear unit (ReLu) is used as an activation function. After the output layer, the SoftMax function is used to normalise the outputs into class probabilities.

Using the open source machine learning library TensorFlow 1.13.2 [107], the accompanying Python Application Programming Interface (API) Keras 2.2.5 [108], and the Python library scikit-learn 0.20.4 [109], a Python script was generated for the training, validation, and testing of various model architectures utilising neural networks with cross-validation. In this script, a fully connected multi-layer perceptron (MLP) is constructed using Keras/TensorFlow that consists of an input layer, a user-specified number of hidden layers each, and an output layer. All but the output layer has a user-specified drop-out to prevent over-fitting [110]. The activation functions in the hidden layers employed rectified-linear units, and the output to class probabilities after the output layer is normalised using the SoftMax function (see Figure 5). For an individual training run, the input data were split into a new pair of training and a validation set for each cross-validation round.

### 4.3. Input Features

Small-molecule compounds, initially identified by their SMILES strings, were represented by a chemical fingerprint, augmented with calculated physico-chemical descriptors. Using the Mordred Python libraries (version 1.2.0) [111], 200 select physico-chemical descriptors were calculated from the SMILES strings. The descriptors were converted to integers and then expressed as bits (*nbits* = 1600). Additionally, Morgan fingerprints [112] with a radius of 6 were calculated and represented as 8192 bits using the implementation in the open source toolkit RDKit (version 2019_03_4). Combining these features, an array of 9792 bits was used as the model input.

Other molecule representations, including simple one-hot encoding, and advanced features, such as the dimensionality-reduced output of a Variational Autoencoder [113], were tested but not pursued as the observed performance was inferior to the above-mentioned approach.

### 4.4. Hyperparameter Tuning

To find the best performing model, different MLP models were generated and trained and their performance was evaluated. The tested model architectures varied in terms of the number of hidden layers (*n_hidden* = 1, 2) and their dimensions. The dimension of the first hidden layer was varied by means of a grid search. The dimension of the second hidden layer was kept constant. Stochastic Gradient Descent was chosen as an optimiser, using momentum with Nesterov’s accelerated gradient [114]. The initial learning rate was set to 1 × 10^−2^, and a minimal learning rate of 1 × 10^−5^ was selected. Momentum was set to constant 0.9. To avoid overfitting, a constant dropout rate of 25% and an L2 regularisation term with a factor of 1 × 10^−4^ were used. In a grid search evaluating the model performance when using different batch sizes (5, 16, 32, 64, 128 and 256), the batch size of 64 resulted in the best model performance, and this setting was then adopted for the final model configuration.

Overall, 76 different model architectures were tested. Using a 75:25 training/validation split of the global training dataset, each model was trained and validated in a 3-fold cross-validation procedure. Using the cross-entropy or Macro F1 Score loss function, the models were trained for 500 epochs. The classification metrics obtained for the model architectures tested are summarised in Tables S3–S7.

### 4.5. In Silico Screening of ZINC Database

A subset of the ZINC15 database [60] comprising compounds over the entire range of available molecular masses and up to a Log*P* of 3.5 (Table S12) was downloaded from the official website. A total of 14.2 million compounds were encoded using Morgan fingerprints and Mordred descriptor, as described above, and used as input for the successfully trained neural network (model m1002a) to predict the bioactivity of compounds against *H. contortus*.

### 4.6. Clustering of Compounds with Predicted Nematocidal Activity

Of the screened subset from the ZINC15 database, 174,539 compounds (1.2%) showed predicted bioactivity against *H. contortus*. This latter group of compounds was then subjected to a clustering step to group together structurally similar compounds, to allow for selection of a small number of distinct and divergent chemotypes for experimental testing. Prior to k-means clustering [81], features were computed for each compound using the Mordred Python libraries [111] and RDKit. The feature set consisted of three components: atom features, bond features, and 200 physico-chemical descriptors. These physico-chemical descriptors were identical to those previously used in the training data preprocessing. The atom and bond features were one-hot encoded, creating feature matrices. To reduce the matrices dimensionality, Principal Component Analysis (PCA) was applied in two stages: first to each individual feature matrix, then to their concatenation. The process generated a combined feature matrix of size 250. Using a custom-trained Variational Autoencoder implemented in PyTorch 1.12 [115], the feature matrix was further reduced to 32 dimensions. This significant reduction in dimensionality proved highly beneficial as it accelerated the *k*-means clustering process. Moreover, the use of a Variational Autoencoder helped preserve the most meaningful compound information during this dimensionality reduction. This approach closely follows [81]. Different values of *k* (=number of clusters) were evaluated using common clustering and evaluation metrics (Silhouette Score, Calinski–Harabsz Index, Davies–Bouldin Index) that were implemented in Python (v.3.8) using the library scikit-learn (v.0.20.4) [109]. To visualise the molecular clusters, the dimensionality of each data point was reduced from 32 to 2 by using the *t*-distributed Stochastic Neighbour Embedding (*t*-SNE) algorithm [116].

### 4.7. Post-Processing and Prioritisation

To aid in the process of sourcing molecules for experimental testing, automated checks for potential patent issues and compound availability were implemented. The website Google Patents (https://patents.google.com/, accessed on 26 March 2025) was queried through its API to receive JSON-formatted patent information for compounds provided as SMILES strings. The information received was processed by fuzzy searching each patent’s title and snippet for the keywords ‘anthelmintic’, ‘anti-parasitic’, ‘haemonchus’, ‘helminth’ and ‘nematode’ with at least 80% identity using Levenshtein distance [117]. If any of the keywords resulted in a hit, the compound was disregarded. In a second step, the MolPort (Riga, Latvia) website (https://www.molport.com/, accessed on 26 March 2025) was queried using the provided API to assess the availability of compounds. The compounds were provided as SMILES strings and the MolPort database was queried for exact matches. Compounds not available for purchase were disregarded. The compounds were appraised chemoinformatically using the software cApp (v.1.5.1) [118]; only compounds without PAINs motifs [119] and meeting Lipinski’s rule-of-five [120] were considered for further medicinal chemical evaluation, and 10 compounds were prioritised.

### 4.8. Assay to Evaluate Bioactivity of Prioritised Compounds

The prioritised compounds were purchased from MolPort and evaluated for their activity by estimating their IC_50_ values and MMIs against xL3s of *H. contortus* (Haecon-5 strain) using an established method [29]. In brief, xL3s were dispensed in 96-well plates (cat. no. 3596; Corning, Corning, NY, USA) at a density of 300 xL3s per well with individual, serially diluted compounds (9-points; 2-fold serial dilution; 50 µM to 0.76 nM), in a final volume of 100 µL of LB* (= sterile lysogenic broth [LB] supplemented with 100 IU/mL penicillin, 100 µg/mL streptomycin and 0.25 µg/mL amphotericin B [Fungizone^®^, Thermo Fisher Scientific, Waltham, MA, USA]). Plates containing xL3s were incubated at 38 °C, 10% (*v*/*v*) CO_2_ and >90% relative humidity. Following 168 h of incubation, the compounds were tested by three independent experiments in triplicate. The xL3s motility and development to fourth-stage larvae (L4s) were measured with reference to two commercial anthelmintics, monepantel (Zolvix^TM^; Elanco, Greenfield, IN, USA) and moxidectin (Cydectin^®^; Virbac, Carros, France). Negative control wells containing 0.25% (*v*/*v*) DMSO were present on all plates. In addition, a set of 20 compounds, predicted as inactive (‘none’) against *H. contortus*, was randomly selected from the ZINC15 database and utilised as negative controls in the validation assay. Compound concentrations were log_10_-transformed and fitted using a variable slope four-parameter equation, constraining the highest value to 100% and employing a least squares (ordinary) fit model as implemented in GraphPad Prism software (v.10.0.1). A one-way analysis of variance (ANOVA) with a Dunnett’s multiple comparison test was used to establish statistically significant differences in larval motility and in development.

Subsequently, the selected compounds evaluated for in vitro activity against larvae were further assessed for their activity against adult females and males of *H. contortus* using an established motility assay [37]. In brief, the compound was dispensed in 24-well plates (cat. no. 3524; Corning) at a concentration of 50 μM in 500 μL of RPMI* (RPMI 1640 medium supplemented with 4 mM *L*-glutamine, 100 U/mL penicillin, 100 µg/mL streptomycin and 0.25 µg/mL amphotericin B). Two positive controls, monepantel and moxidectin, were each prepared in the same manner, and a negative control containing 0.25% (*v*/*v*) DMSO was included on all plates. Three adult females or males were added to each of the wells containing either the test compound or a control compound and then incubated at 38 °C, 10% (*v*/*v*) CO_2_ and >90% relative humidity for 24 h. The compounds were tested in triplicate. A video recording (30 sec) of each well was taken at 0 h, 3 h, 6 h, 12 h and 24 h during the entire incubation period to assess the reduction in worm motility—scored as 3 (‘good’), 2 (‘low’), 1 (‘very low’) or 0 (‘none’). For each test or control compound, the motility scores for each of the triplicate wells were calculated, normalised with reference to the negative control (100% motility), and recorded as a percentage. A two-way ANOVA with Dunnett’s multiple comparison test was used to establish statistically significant differences against the DMSO control in worm motility for each time point.

### 4.9. Method to Assess Cytotoxicity of Prioritised Compounds

The cytotoxicity of the prioritised compounds **3**, **4** and **6** on human hepatoma (HepG2) cells was evaluated as described previously [121]. In brief, HepG2 cells were first cultured (in an incubator at 5% (*v*/*v*) CO_2_, 37 °C and >90% humidity) in Dulbecco’s modified Eagle’s medium (DMEM), supplemented with 5% (*v*/*v*) foetal bovine serum (FBS). The test compounds were serially diluted (10-points; 2-fold serial dilution; top concentration of 50 μM) in DMEM plus 10% (*v*/*v*) FBS, adjusted to a DMSO concentration of 0.5% (*v*/*v*) and arrayed across a 384-well plate. Bortezomib (10 μM) was used as a positive control, and 0.5% DMSO was used as a negative control. HepG2 cells (1 × 10^3^ cells per 50 μL of DMEM plus 10% FBS) were then seeded into wells of the assay plate; the plates were then incubated in 5% (*v*/*v*) CO_2_, 37 °C and >90% humidity for 48 h. Subsequently, cell proliferation was assessed using CellTiter-Glo (Promega, Fitchburg, WI, USA) and normalised against the negative controls and expressed as a percentage. All compounds were tested in duplicate. The half-maximal cytotoxic (CC_50_) values were calculated using the software Dotmatics (v.5.3) and Spotfire (v.7.11.1) employing a non-linear regression four-parameter fit analysis.

## 5. Conclusions

Our deep learning-assisted approach harnessed an extensive body of in-house bioactivity data from previous screenings of *H. contortus*, supplemented with activity data from the literature to expedite the discovery of novel anthelmintics. Through the application of machine learning, the large number of inactive compounds that typically have no further utility in conventional drug discovery programmes against a chosen target could be put to further use, unveiling their potential through advanced computational analysis. Different machine learning methodologies were tested, and the application of multi-layer perceptrons for bioactivity classification showed acceptable performance. In contrast, attempts to construct regression models to predict numerical activity data did not yield satisfactory results in our hands. As demonstrated by the confusion matrix, the classification model generated in this study achieves a good distinction of ‘active’ from ‘other’ (i.e., ‘weakly active’ and ‘none’) compounds. The separation of ‘weakly active’ compounds from inactive (‘none’) ones, however, is less reliable. This model was integral in screening >14 million compounds to identify novel nematostatic and nematocidal agents, leading to the evaluation of 10 compounds with predicted activity. All of these compounds inhibited *H. contortus* and two compounds emerged as significant leads. Moving forward, in-depth investigations of the modes/mechanisms of action of these lead compounds, supported by in silico docking studies and further pharmacological profiling, will be instrumental in progressing these compounds from the bench to the market. Our findings provide a new avenue for the development of effective anthelmintics and underline the important role of computational tools in complementing traditional anthelmintic discovery approaches. This advance could accelerate discovery efforts, focused on sustaining the control of parasitic helminths that adversely impact animal and human health worldwide.

As the deployment of machine learning-based approaches in future drug discovery efforts will undoubtedly increase, it seems prudent to work towards publicly available, unified data collections to support the application of such methodologies. To a large extent, the data organisation and data availability in the drug discovery realm is still locked in a situation characterised by media discontinuity and disparate formats, rendering much of the medicinal chemistry and bioactivity information inaccessible for direct use. In order to achieve the best possible efficiency for contemporary approaches and to transform the current situation, more work needs to be undertaken to assemble relevant data into curated accessible databases. Accordingly, with this study, we also introduce an online resource for the community *en route* to a unified database for small-molecule compounds with antiparasitic properties.

## Figures and Tables

**Table 1:** Classification criteria defined and applied in this study to covert numerical assay data into activity labels.

Activity Label	Wiggle Index	Viability	Reduction	EC_50_	MIC_75_
**active**	*x* < 0.25	*x* < 20%	*x* > 80%	*x* < 50 µM	*x* < 1 µg/mL
**weakly active**	0.25 ≤ *x* < 0.5	20% ≤ *x* < 50%	80% ≥ *x* > 50%	50 µM ≤ *x* < 100 µM	1 µg/mL ≤ *x* < 10 µg/mL
**none**	0.5 ≤ *x*	50% ≤ *x*	50% ≥ *x*	100 µM ≤ *x*	10 µg/mL ≤ *x*

**Table 2:** Small-molecule bioactivity data used for training and validation [63].

Library/Compound Family	*N*	Reference
Open Scaffolds	14,464	[26]
Pathogen Box	400	[61]
1-methyl-1H-pyrazole-5-carboxamides	64	[64]
Tolfenpyrad derivatives	57	[65]
pyrrolidine-oxadiazoles	57	[66]
1-methyl-1H-pyrazole-5-carboxamides	30	[67]
Monepantel derivatives	28	[68]
Tetrahydroquinoxalines	1	[69]
Selenophenes, thiophenes	12	[70]
Diarylamidines	6	[71]
Milbemycine derivatives	4	[72]
Anthelmintics (rafoxanide, naphthalophos, nitroxynil, disophenol)	4	[16]
Phosphoethanolamine methyltransferases inhibitors	3	[73]
*p*-amino-phenethyl-*m*-trifluoromethylphenyl piperazine	3	[74]
Anthelmintics (derquantel, abamectin)	2	[75]
Anthelmintics (abamectin, benzimidazole)	2	[76]
Fromiamycalin, halaminol A	2	[77]
Anthelmintic (closantel)	1	[78]
Phenothiazine	1	[79]
Deguelin	1	[27]
Anthelmintic (eprinomectin)	1	[80]
Anthelmintic (albendazole, monepantel, morantel, moxidectin, thiabendazole)	5	in-house data
**Total**	**15,162**	

**Table 3:** Splitting of the curated dataset into a global training and a global test dataset.

Label	Training		Test		Total	
	#	Fraction	#	Fraction	#	Fraction
None	11,005	0.902	2643	0.894	13,648	0.900
Weakly active	1081	0.089	272	0.092	1353	0.089
Active	121	0.010	40	0.014	161	0.011
(Combined active)	1202	0.098	314	0.106	1516	0.100
**Total**	**12,207**	**0.81**	**2955**	**0.19**	**15,162**	**1.0**

**Table 4:** Summary of model architectures generated in grid search series and parameters of best models from each series.

	Tested Models			Best Model Performance on Test Set		
Series	No of Models	*n_hidden*	*dim_hidden*	*dim_hidden*	Precision ‘Active’	Recall ‘Active’
series_1001	39	1	5–195	100	0.816	0.756
series_1002	19	2	5–95/5	75/5	0.825	0.805
series_1003	9	2	100–900/10	500/10	0.821	0.780
series_1004	9	2	100–900/50	600/50	0.811	0.732
series_2001	39	1	5–195	185	0.789	0.732

**Table 5:** Predicted nematocidal activity of ZINC15 subset subjected to in silico screening.

	Predicted Bioactivity Against *H. contortus*		
No of Compounds	Active	Weakly Active	None
14,154,291	174,539	12,922	13,966,830
	1.2%	0.09%	98.7%

**Table 6:** The bioactivity of 10 prioritised small compounds with predicted activity against *Haemonchus contortus*. The potencies of the inhibitory effect of compounds against exsheathed third-stage larvae (xL3s) of *H. contortus* with reference to two control compounds (monepantel and moxidectin) were assessed. The half-maximal inhibitory concentration (IC_50_; µM) on the larval motility and development, maximal inhibitions (MI; %), and the induced abnormal phenotypes in affected larvae were assessed after 168 h of exposure. For compound names and identifiers, see Table S9.

Compound	Structure	Larval Motility IC_50_ μM ^a^ (MI; %)	Larval Development IC_50_ μM ^a^ (MI; %)	Abnormal Phenotype ^b^ (Exhibited at 50 µM; %)
**1**	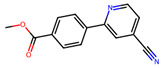	6.2 ± 0.3 (50)	>50	*Cur*, *Evi* (20)
**2**	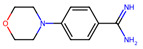	1.8 ± 1.4 (53)	>50	nil
**3**	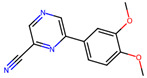	7.9 ± 1.9 (54)	37.1 (100)	*Evi* (80)
**4**	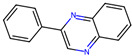	5.8 ± 4.1 (90)	>50	nil
**5**	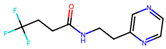	37.5 ± 2.5 (65)	>50	nil
**6**	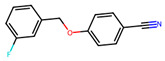	7.4 ± 9.4 (100)	23.6 (100)	*Evi* (100)
**7**	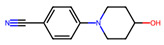	2.1 ± 3.5 (70)	>50	nil
**8**	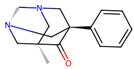	0.8 ± 1.6 (42)	>50	nil
**9**	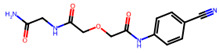	4.1 ± 21.4 (46)	>50	nil
**10**	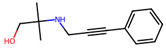	0.8 ± 1.2 (50)	>50	nil
Monepantel		0.2 ± 0.002 (100)	0.4 (100)	*Coi* (100)
Moxidectin		0.8 ± 0.4 (75)	45.0 (100)	nil

^a^ IC_50_ calculated from three independent assays in triplicate. ^b^ Phenotypes: curved (*Cur*), evisceration (*Evi*), coiled (*Coi*).

## Data Availability

The code of the computational parts of the study is available on Zenodo (DOI 10.5281/zenodo.14511148). The dataset of 15,162 compounds with classified bioactivity labels (see Table 2) assembled within this study was deposited with Zenodo (DOI 10.5281/zenodo.10929251). Bioactivity data extracted from the scientific literature and annotated with the bioactivity labels as described in this study are available in the Antiparasitics DB at https://www.antiparasiticsdb.org (accessed on 26 March 2025).

## Supplementary Materials

The supporting information can be downloaded at: https://www.mdpi.com/article/10.3390/ijms26073134/s1.
