# Supplementary material for: Prediction and Prioritisation of Novel Anthelmintic Candidates from Public Databases Using Deep Learning and Available Bioactivity Data Sets
Source: Int J Mol Sci. 2025 Mar 28;26(7):3134. doi: 10.3390/ijms26073134 (PMC11988817; doi:10.3390/ijms26073134)

Supporting information

Prediction and prioritisation of novel anthelmintic candidates from public databases by using deep learning and available bioactivity data sets

Aya C. Taki[1], Louis Kapp[1,2] , Ross S. Hall[1], Joseph J. Byrne[1], Brad E. Sleebs[1,3], Bill C. H. Chang[1], Robin B. Gasser[1,*] and Andreas Hofmann[1,4,*]

[1] Melbourne Veterinary School, Faculty of Science, The University of Melbourne, Parkville, Victoria 3010, Australia
[2] Institute of Cognitive Science, University of Osnabrück, 49090 Osnabrück, Germany
[3] Walter and Eliza Hall Institute of Medical Research, Parkville, Victoria 3052, Australia
[4] Max Rubner-Institut, Federal Research Institute of Nutrition and Food, 95326 Kulmbach, Germany

[*] Corresponding authors
E-mail: robinbg@unimelb.edu.au (RBG), a.hofmann@structuralchemistry.org (AH)

Table S1. Results of machine learning using scikit-learn support vector machine regression.

Table S2. Results of deep learning using a Keras/TensorFlow MLP for regression.

Table S3. Classification metrics of model architectures tested in series 1001.

Table S4. Classification metrics of model architectures tested in series 1002.

Table S5. Classification metrics of model architectures tested in series 1003.

Table S6. Classification metrics of model architectures tested in series 1004.

Table S7. Classification metrics of model architectures tested in series 2001.

Table S8. The 123 compounds used for the selection of prioritised set for experimental validation.

Table S9. Chemical identifiers of 10 small-molecule compounds predicted to be ‘active’ against *Haemonchus contortus* and two commercial anthelmintic compounds (monepantel and moxidectin) used as positive controls.

Table S10. Chemical identifiers of 20 small-molecule compounds randomly selected from the ZINC15 database.

Table S11. Similarity of compounds 3 and 6 with compounds that possess antiparasitic activity.

Table S12. Compound library assembled as a subset of the ZINC15 database and used in the *in silico* screening.

Figure S1. The dose-response assessment of *in vitro* motility inhibition of 10 prioritised small molecules from ZINC database, predicted to be 'active' against *Haemonchus contortus*.

Figure S2. The dose-response assessment of *in vitro* motility inhibition of 20 randomly chosen small molecules from ZINC database, predicted to be inactive ('none') against *Haemonchus contortus*.

Table S1. Results of machine learning using scikit-learn support vector machine regression.

	gamma	***cost***	***R*² training**	***R*² validation**	***R*² test**
series_002	1000	0.1	0.664	0	0
series_002	100	0.1	0.663	0	0
series_002	10	0.1	0.663	0	0
series_001	1	0.1	0.663	0	0.006
series_001	0.1	0.1	0.664	0.001	0.005
series_001	0.01	0.1	0.311	0.006	0.012
series_001	0.001	0.1	0.084	0.005	0.008
series_001	0.0001	0.1	0.052	0.004	0.009
series_002	1000	1	0.873	0	0
series_002	100	1	0.874	0	0
series_002	10	1	0.875	0	0
series_001	1	1	0.876	0	0.014
series_001	0.1	1	0.873	0	0.014
series_001	0.01	1	0.699	0.005	0.009
series_001	0.001	1	0.211	0.007	0.009
series_001	0.0001	1	0.079	0.005	0.01
series_002	1000	10	0.995	0	0
series_002	100	10	0.995	0	0
series_002	10	10	0.995	0	0
series_001	1	10	0.996	0	0.018
series_001	0.1	10	0.995	0	0.011
series_001	0.01	10	0.993	0.006	0.003
series_001	0.001	10	0.482	0.005	0.002
series_001	0.0001	10	0.199	0.005	0.006

Data fitting using support vector machine regression was done by using the scikit-learn SVR module and bioactivity data (N_{total} = 402) obtained for the Pathogen Box (N = 400) and positive controls (N = 2). A radial basis function kernel was used and the *gamma* ($10^{-4} – 10^{3}$) and *cost* (0.1 – 10) parameters were varied in a grid search to optimise the fit. Ten-fold cross validation was carried by testing each set of chosen parameters with different training (75%) and test (25%) sets obtained from the supplied training data set at the beginning of each run. Root mean square error and correlation R^2 between experimental and predicted values were monitored.

Whereas a reasonable correlation could be achieved in the training data set (best R^2 = 0.966), prediction of the validation and training data was not possible (best R^2 = 0 and 0.018, respectively).

Table S2. Results of deep learning using a Keras/TensorFlow MLP for regression.

	initial *learning rate*	*momentum*	*dropout rate* input / hidden	*n_hidden*	*dim_hidden*	*batch_size*	Training R^2	Validation R^2	Test R^2
series_001	0.01	0.9	0.1 / 0.25	1	25	1000	0.838	0.004	0.006
	0.01	0.9	0.1 / 0.25	1	30	1000	0.842	0.005	0.003
	0.01	0.9	0.1 / 0.25	1	35	1000	0.847	0.005	0.005
	0.01	0.9	0.1 / 0.25	1	40	1000	0.853	0.006	0.003
	0.01	0.9	0.1 / 0.25	1	45	1000	0.857	0.005	0.005
	0.01	0.9	0.1 / 0.25	1	50	1000	0.857	0.005	0.004
	0.01	0.9	0.1 / 0.25	1	55	1000	0.86	0.005	0.005
	0.01	0.9	0.1 / 0.25	1	60	1000	0.861	0.005	0.005
	0.01	0.9	0.1 / 0.25	1	65	1000	0.865	0.006	0.004
	0.01	0.9	0.1 / 0.25	1	70	1000	0.864	0.005	0.005
	0.01	0.9	0.1 / 0.25	1	75	1000	0.865	0.006	0.003
	0.01	0.9	0.1 / 0.25	1	80	1000	0.866	0.005	0.006
	0.01	0.9	0.1 / 0.25	1	85	1000	0.867	0.005	0.003
	0.01	0.9	0.1 / 0.25	1	90	1000	0.869	0.005	0.005
	0.01	0.9	0.1 / 0.25	1	95	1000	0.868	0.004	0.004
	0.01	0.9	0.1 / 0.25	1	100	1000	0.869	0.005	0.006
series_002	0.01	0.9	0.1 / 0.25	1	25	5000	0.795	0.004	0.006
	0.01	0.9	0.1 / 0.25	1	30	5000	0.805	0.005	0.005
	0.01	0.9	0.1 / 0.25	1	35	5000	0.814	0.006	0.006
	0.01	0.9	0.1 / 0.25	1	40	5000	0.817	0.005	0.002
	0.01	0.9	0.1 / 0.25	1	45	5000	0.821	0.005	0.003
	0.01	0.9	0.1 / 0.25	1	50	5000	0.823	0.006	0.005
	0.01	0.9	0.1 / 0.25	1	55	5000	0.828	0.005	0.002
	0.01	0.9	0.1 / 0.25	1	60	5000	0.828	0.005	0.001
	0.01	0.9	0.1 / 0.25	1	65	5000	0.829	0.005	0.005
	0.01	0.9	0.1 / 0.25	1	70	5000	0.828	0.006	0.005
	0.01	0.9	0.1 / 0.25	1	75	5000	0.832	0.006	0.003
	0.01	0.9	0.1 / 0.25	1	80	5000	0.835	0.007	0.005
	0.01	0.9	0.1 / 0.25	1	85	5000	0.837	0.005	0.005
	0.01	0.9	0.1 / 0.25	1	90	5000	0.841	0.006	0.005
	0.01	0.9	0.1 / 0.25	1	95	5000	0.839	0.005	0.004
	0.01	0.9	0.1 / 0.25	1	100	5000	0.839	0.004	0.005
series_003	0.01	0.9	0.1 / 0.25	2	25, 25	5000	0.958	0.004	0.004
	0.01	0.9	0.1 / 0.25	2	30, 30	5000	0.961	0.005	0.004
	0.01	0.9	0.1 / 0.25	2	35, 35	5000	0.958	0.006	0.005
	0.01	0.9	0.1 / 0.25	2	40, 40	5000	0.966	0.005	0.010
	0.01	0.9	0.1 / 0.25	2	45, 45	5000	0.966	0.006	0.006
	0.01	0.9	0.1 / 0.25	2	50, 50	5000	0.968	0.005	0.007
	0.01	0.9	0.1 / 0.25	2	55, 55	5000	0.967	0.003	0.002
	0.01	0.9	0.1 / 0.25	2	60, 60	5000	0.963	0.005	0.003
	0.01	0.9	0.1 / 0.25	2	65, 65	5000	0.97	0.005	0.009
	0.01	0.9	0.1 / 0.25	2	70, 70	5000	0.97	0.004	0.005
	0.01	0.9	0.1 / 0.25	2	75, 75	5000	0.974	0.005	0.003
	0.01	0.9	0.1 / 0.25	2	80, 80	5000	0.971	0.005	0.002
	0.01	0.9	0.1 / 0.25	2	85, 85	5000	0.975	0.006	0.005
	0.01	0.9	0.1 / 0.25	2	90, 90	5000	0.97	0.006	0.004

	0.01	0.9	0.1 / 0.25	2	95, 95	5000	0.975	0.004	0.007
	0.01	0.9	0.1 / 0.25	2	100, 100	5000	0.976	0.005	0.003
series_004	0.01	0.9	0.2 / 0.5	1	1024	5000	0.996	0.002	0.005
	0.01	0.9	0.2 / 0.5	1	2048	5000	0.997	0.006	0
	0.01	0.9	0.2 / 0.5	1	4096	5000	0.998	0.009	0.002
	0.01	0.9	0.2 / 0.5	1	8192	5000	0.998	0.010	0.003
	0.01	0.9	0.2 / 0.5	1	16384	5000	0.998	0.006	0.009
series_005	0.01	0.9	0.2 / 0.5	2	1024, 1024	5000	0.994	0.002	0.001
	0.01	0.9	0.2 / 0.5	2	2048, 2048	5000	0.995	0.008	0
	0.01	0.9	0.2 / 0.5	2	4096, 4096	5000	0.995	0.007	0.001
	0.01	0.9	0.2 / 0.5	2	8192 8192	5000	0.996	0.006	0.003
	0.01	0.9	0.2 / 0.5	2	16384, 16384	5000	0.996	0.006	0
series_006	0.01	0.9	0.2 / 0.5	3	1024 all	5000	0.987	0.002	0.004
	0.01	0.9	0.2 / 0.5	3	2048 all	5000	0.986	0.007	0.005
	0.01	0.9	0.2 / 0.5	3	4096 all	5000	0.987	0.004	0.005
	0.01	0.9	0.2 / 0.5	3	8192 all	5000	0.990	0.006	0.005
	0.01	0.9	0.2 / 0.5	3	16384 all	5000	-	-	-
series_007	0.1	0.9	0.2 / 0.5	1	1024	5000	0.994	0.004	0.001
	0.1	0.9	0.2 / 0.5	1	2048	5000	0.983	0.004	0
	0.1	0.9	0.2 / 0.5	1	4096	5000	0	0	0
	0.1	0.9	0.2 / 0.5	1	8192	5000	0	0	0
	0.1	0.9	0.2 / 0.5	1	16384	5000	0	0	0
series_008	0.1	0.9	0.2 / 0.5	2	1024, 1024	5000	0.992	0.007	0.025
	0.1	0.9	0.2 / 0.5	2	2048, 2048	5000	0.987	0.001	0.014
	0.1	0.9	0.2 / 0.5	2	4096, 4096	5000	0.982	0.006	0.004
	0.1	0.9	0.2 / 0.5	2	8192 8192	5000	-	-	-
	0.1	0.9	0.2 / 0.5	2	16384, 16384	5000	-	-	-
series_009	0.01	0.75	0.2 / 0.5	1	1024	5000	0.992	0.007	0.004
	0.01	0.75	0.2 / 0.5	1	2048	5000	0.993	0.007	0.002
	0.01	0.75	0.2 / 0.5	1	4096	5000	0.994	0.007	0.002
	0.01	0.75	0.2 / 0.5	1	8192	5000	0.995	0.007	0.020
	0.01	0.75	0.2 / 0.5	1	16384	5000	0.995	0.012	0.003

Fully-connected multilayer perceptrons were constructed using Keras/TensorFlow consisting of an input layer with a dropout rate of 10%, either one or two hidden layers each with a dropout rate of 25% and an output layer. The number of nodes in the hidden layer was varied between 25 and 100 and batch sizes of either 1000 or 5000 were used. The *l2* kernel regularisation with *l2* = 0.016 was used. The learning rate was dynamically decreased. The epochs parameter was 1000 for all runs. Ten-fold cross validation was carried by testing each set of chosen parameters with different training (75%) and test (25%) sets obtained from the supplied training data set at the beginning of each run. Root mean square error and correlation R^2 between experimental and predicted values were monitored.

A total of nine series were computed using bioactivity data (N_{total} = 14866) obtained for the Pathogen Box (N = 400), the Open Scaffolds collection (N = 14464) and positive controls (N = 2). While a good correlation could be achieved in the training data set (best R^2 = 0.998), prediction of the validation and training data was not possible (best R^2 = 0.012 and 0.025, respectively).

Table S3. Classification metrics of model architectures tested in series 1001.

Training / validation: 75 / 25
No of hidden layers: 1 (*dim_hidden1* = 5 – 195)
CV: 3-fold
Loss function: Categorical cross entropy

						None				Weakly active				Active				Combined active			
**Run**	***dim_hidden1***	**Training accuracy**		**Validation accuracy**		**True positive**		**False positive**		**True positive**		**False positive**		**True positive**		**False positive**		**True positive**		**False positive**	
		**mean**	**std**	**mean**	**std**	**mean**	**std**	**mean**	**std**	**mean**	**std**	**mean**	**std**	**mean**	**std**	**mean**	**std**	**mean**	**std**	**mean**	**std**
1	5	0.95	0	0.9	0	0.99	0.00	0.01	0.00	0.02	0.00	0.98	0.00	0.23	0.00	0.77	0.00	0.09	0.00	0.91	0.00
2	10	0.96	0	0.89	0	0.98	0.00	0.02	0.00	0.04	0.00	0.96	0.00	0.35	0.00	0.65	0.00	0.10	0.00	0.90	0.00
3	15	0.97	0	0.89	0	0.98	0.00	0.02	0.00	0.01	0.00	0.99	0.00	0.58	0.00	0.42	0.00	0.08	0.00	0.92	0.00
4	20	0.98	0	0.9	0	0.99	0.00	0.01	0.00	0.00	0.00	1.00	0.00	0.71	0.00	0.29	0.00	0.09	0.00	0.91	0.00
5	25	0.98	0	0.9	0	0.99	0.00	0.01	0.00	0.02	0.00	0.98	0.00	0.68	0.00	0.32	0.00	0.10	0.00	0.90	0.00
6	30	0.99	0	0.89	0	0.98	0.00	0.02	0.00	0.01	0.00	0.99	0.00	0.68	0.00	0.32	0.00	0.10	0.00	0.90	0.00
7	35	0.98	0	0.9	0	0.99	0.00	0.01	0.00	0.01	0.00	0.99	0.00	0.68	0.00	0.32	0.00	0.09	0.00	0.91	0.00
8	40	0.99	0	0.89	0	0.98	0.00	0.02	0.00	0.01	0.00	0.99	0.00	0.74	0.00	0.26	0.00	0.10	0.00	0.90	0.00
9	45	0.99	0	0.89	0	0.98	0.00	0.02	0.00	0.01	0.00	0.99	0.00	0.71	0.00	0.29	0.00	0.10	0.00	0.90	0.00
10	50	0.99	0	0.9	0	0.99	0.00	0.01	0.00	0.02	0.00	0.98	0.00	0.68	0.00	0.32	0.00	0.10	0.00	0.90	0.00
11	55	0.99	0	0.89	0	0.98	0.00	0.02	0.00	0.02	0.00	0.98	0.00	0.71	0.00	0.29	0.00	0.10	0.00	0.90	0.00
12	60	1	0	0.9	0	0.99	0.00	0.01	0.00	0.02	0.00	0.98	0.00	0.68	0.00	0.32	0.00	0.10	0.00	0.90	0.00
13	65	1	0	0.9	0	0.99	0.00	0.01	0.00	0.02	0.00	0.98	0.00	0.71	0.00	0.29	0.00	0.10	0.00	0.90	0.00
14	70	1	0	0.9	0	0.99	0.00	0.01	0.00	0.02	0.00	0.98	0.00	0.71	0.00	0.29	0.00	0.10	0.00	0.90	0.00
15	75	1	0	0.9	0	0.99	0.00	0.01	0.00	0.02	0.00	0.98	0.00	0.71	0.00	0.29	0.00	0.11	0.00	0.89	0.00
16	80	0.99	0	0.9	0	0.99	0.00	0.01	0.00	0.01	0.00	0.99	0.00	0.58	0.00	0.42	0.00	0.08	0.00	0.92	0.00
17	85	0.99	0	0.9	0	0.99	0.00	0.01	0.00	0.01	0.00	0.99	0.00	0.65	0.00	0.35	0.00	0.08	0.00	0.92	0.00
18	90	1	0	0.9	0	0.99	0.00	0.01	0.00	0.01	0.00	0.99	0.00	0.61	0.00	0.39	0.00	0.09	0.00	0.91	0.00
19	95	1	0	0.9	0	0.99	0.00	0.01	0.00	0.02	0.00	0.98	0.00	0.74	0.00	0.26	0.00	0.10	0.00	0.90	0.00
20	100	0.99	0	0.9	0	0.99	0.00	0.01	0.00	0.02	0.00	0.98	0.00	0.77	0.00	0.23	0.00	0.11	0.00	0.89	0.00
21	105	1	0	0.9	0	0.99	0.00	0.01	0.00	0.01	0.00	0.99	0.00	0.71	0.00	0.29	0.00	0.10	0.00	0.90	0.00

22	110	1	0	0.9	0	0.99	0.00	0.01	0.00	0.01	0.00	0.99	0.00	0.74	0.00	0.26	0.00	0.10	0.00	0.90	0.00
23	115	1	0	0.9	0	0.99	0.00	0.01	0.00	0.01	0.00	0.99	0.00	0.68	0.00	0.32	0.00	0.10	0.00	0.90	0.00
24	120	1	0	0.9	0	0.99	0.00	0.01	0.00	0.01	0.00	0.99	0.00	0.74	0.00	0.26	0.00	0.10	0.00	0.90	0.00
25	125	1	0	0.9	0	0.99	0.00	0.01	0.00	0.02	0.00	0.98	0.00	0.71	0.00	0.29	0.00	0.10	0.00	0.90	0.00
26	130	1	0	0.9	0	0.99	0.00	0.01	0.00	0.01	0.00	0.99	0.00	0.68	0.00	0.32	0.00	0.10	0.00	0.90	0.00
27	135	1	0	0.9	0	0.99	0.00	0.01	0.00	0.02	0.00	0.98	0.00	0.71	0.00	0.29	0.00	0.10	0.00	0.90	0.00
28	140	1	0	0.9	0	0.99	0.00	0.01	0.00	0.01	0.00	0.99	0.00	0.61	0.00	0.39	0.00	0.09	0.00	0.91	0.00
29	145	1	0	0.9	0	0.99	0.00	0.01	0.00	0.02	0.00	0.98	0.00	0.71	0.00	0.29	0.00	0.10	0.00	0.90	0.00
30	150	1	0	0.9	0	0.99	0.00	0.01	0.00	0.02	0.00	0.98	0.00	0.68	0.00	0.32	0.00	0.10	0.00	0.90	0.00
31	155	1	0	0.9	0	0.99	0.00	0.01	0.00	0.02	0.00	0.98	0.00	0.74	0.00	0.26	0.00	0.10	0.00	0.90	0.00
32	160	1	0	0.9	0	0.99	0.00	0.01	0.00	0.01	0.00	0.99	0.00	0.71	0.00	0.29	0.00	0.10	0.00	0.90	0.00
33	165	1	0	0.9	0	0.99	0.00	0.01	0.00	0.01	0.00	0.99	0.00	0.65	0.00	0.35	0.00	0.09	0.00	0.91	0.00
34	170	1	0	0.9	0	0.99	0.00	0.01	0.00	0.02	0.00	0.98	0.00	0.68	0.00	0.32	0.00	0.10	0.00	0.90	0.00
35	175	1	0	0.9	0	0.99	0.00	0.01	0.00	0.02	0.00	0.98	0.00	0.74	0.00	0.26	0.00	0.11	0.00	0.89	0.00
36	180	1	0	0.9	0	0.99	0.00	0.01	0.00	0.02	0.00	0.98	0.00	0.68	0.00	0.32	0.00	0.10	0.00	0.90	0.00
37	185	1	0	0.9	0	0.99	0.00	0.01	0.00	0.01	0.00	0.99	0.00	0.65	0.00	0.35	0.00	0.09	0.00	0.91	0.00
38	190	1	0	0.9	0	1.00	0.00	0.00	0.00	0.01	0.00	0.99	0.00	0.68	0.00	0.32	0.00	0.10	0.00	0.90	0.00
39	195	1	0	0.9	0	0.99	0.00	0.01	0.00	0.01	0.00	0.99	0.00	0.68	0.00	0.32	0.00	0.10	0.00	0.90	0.00

Table S4. Classification metrics of model architectures tested in series 1002.

Training / validation: 75 / 25
No of hidden layers: 2 (*dim_hidden1* = 5 – 95, *dim_hidden2* = 5)
CV: 3-fold
Loss function: Categorical cross entropy

						None				Weakly active				Active				Combined active			
**Run**	***dim_hidden1***	**Training accuracy**		**Validation accuracy**		**True positive**		**False positive**		**True positive**		**False positive**		**True positive**		**False positive**		**True positive**		**False positive**	
		**mean**	**std**	**mean**	**std**	**mean**	**std**	**mean**	**std**	**mean**	**std**	**mean**	**std**	**mean**	**std**	**mean**	**std**	**mean**	**std**	**mean**	**std**
1	5	0.9	0	0.9	0	1.00	0.00	0.00	0.00	0.00	0.00	1.00	0.00	0.00	0.00	1.00	0.00	0.00	0.00	1.00	0.00
2	10	0.93	0	0.88	0	0.97	0.00	0.03	0.00	0.03	0.00	0.97	0.00	0.13	0.00	0.87	0.00	0.10	0.00	0.90	0.00
3	15	0.93	0	0.89	0	0.98	0.00	0.02	0.00	0.02	0.00	0.98	0.00	0.65	0.00	0.35	0.00	0.10	0.00	0.90	0.00
4	20	0.97	0	0.88	0	0.97	0.00	0.03	0.00	0.04	0.00	0.96	0.00	0.39	0.00	0.61	0.00	0.12	0.00	0.88	0.00
5	25	0.97	0	0.89	0	0.98	0.00	0.02	0.00	0.01	0.00	0.99	0.00	0.74	0.00	0.26	0.00	0.10	0.00	0.90	0.00
6	30	0.92	0	0.9	0	0.99	0.00	0.01	0.00	0.01	0.00	0.99	0.00	0.26	0.00	0.74	0.00	0.09	0.00	0.91	0.00
7	35	0.97	0	0.89	0	0.99	0.00	0.01	0.00	0.03	0.00	0.97	0.00	0.35	0.00	0.65	0.00	0.11	0.00	0.89	0.00
8	40	0.97	0	0.89	0	0.98	0.00	0.02	0.00	0.04	0.00	0.96	0.00	0.10	0.00	0.90	0.00	0.11	0.00	0.89	0.00
9	45	0.96	0	0.87	0	0.96	0.00	0.04	0.00	0.03	0.00	0.97	0.00	0.23	0.00	0.77	0.00	0.11	0.00	0.89	0.00
10	50	0.97	0	0.88	0	0.97	0.00	0.03	0.00	0.04	0.00	0.96	0.00	0.19	0.00	0.81	0.00	0.13	0.00	0.87	0.00
11	55	0.98	0	0.89	0	0.98	0.00	0.02	0.00	0.03	0.00	0.97	0.00	0.29	0.00	0.71	0.00	0.11	0.00	0.89	0.00
12	60	0.97	0	0.9	0	0.99	0.00	0.01	0.00	0.03	0.00	0.97	0.00	0.19	0.00	0.81	0.00	0.10	0.00	0.90	0.00
13	65	0.97	0	0.89	0	0.98	0.00	0.02	0.00	0.03	0.00	0.97	0.00	0.19	0.00	0.81	0.00	0.10	0.00	0.90	0.00
14	70	0.99	0	0.89	0	0.98	0.00	0.02	0.00	0.02	0.00	0.98	0.00	0.74	0.00	0.26	0.00	0.10	0.00	0.90	0.00
15	75	0.98	0	0.9	0	0.99	0.00	0.01	0.00	0.03	0.00	0.97	0.00	0.77	0.00	0.23	0.00	0.11	0.00	0.89	0.00
16	80	0.99	0	0.89	0	0.98	0.00	0.02	0.00	0.02	0.00	0.98	0.00	0.42	0.00	0.58	0.00	0.11	0.00	0.89	0.00
17	85	0.98	0	0.89	0	0.98	0.00	0.02	0.00	0.02	0.00	0.98	0.00	0.65	0.00	0.35	0.00	0.09	0.00	0.91	0.00
18	90	0.99	0	0.89	0	0.98	0.00	0.02	0.00	0.03	0.00	0.97	0.00	0.71	0.00	0.29	0.00	0.11	0.00	0.89	0.00
19	95	0.99	0	0.89	0	0.98	0.00	0.02	0.00	0.03	0.00	0.97	0.00	0.48	0.00	0.52	0.00	0.11	0.00	0.89	0.00

Table S5. Classification metrics of model architectures tested in series 1003.

Training / validation: 75 / 25
No of hidden layers: 2 (*dim_hidden1* = 100 – 900, *dim_hidden2* = 10)
CV: 3-fold
Loss function: Categorical cross entropy

						None				Weakly active				Active			
**Run**	***dim_hidden1***	**Training accuracy**		**Validation accuracy**		**True positive**		**False positive**		**True positive**		**False positive**		**True positive**		**False positive**	
		**mean**	**std**	**mean**	**std**	**mean**	**std**	**mean**	**std**	**mean**	**std**	**mean**	**std**	**mean**	**std**	**mean**	**std**
1	100	0.94	0.00	0.88	0.00	0.98	0.00	0.02	0.00	0.03	0.00	0.97	0.00	0.00	0.00	1.00	0.00
2	200	0.95	0.00	0.90	0.00	0.99	0.00	0.01	0.00	0.01	0.00	0.99	0.00	0.65	0.00	0.35	0.00
3	300	0.96	0.00	0.89	0.00	0.98	0.00	0.02	0.00	0.03	0.00	0.97	0.00	0.65	0.00	0.35	0.00
4	400	0.98	0.00	0.90	0.00	0.99	0.00	0.01	0.00	0.02	0.00	0.98	0.00	0.61	0.00	0.39	0.00
5	500	0.98	0.00	0.89	0.00	0.98	0.00	0.02	0.00	0.03	0.00	0.97	0.00	0.74	0.00	0.26	0.00
6	600	0.98	0.00	0.90	0.00	0.99	0.00	0.01	0.00	0.01	0.00	0.99	0.00	0.61	0.00	0.39	0.00
7	700	0.99	0.00	0.89	0.00	0.98	0.00	0.02	0.00	0.03	0.00	0.97	0.00	0.71	0.00	0.29	0.00
8	800	0.98	0.00	0.89	0.00	0.98	0.00	0.02	0.00	0.02	0.00	0.98	0.00	0.61	0.00	0.39	0.00
9	900	0.99	0.00	0.90	0.00	0.99	0.00	0.01	0.00	0.01	0.00	0.99	0.00	0.71	0.00	0.29	0.00

Table S6. Classification metrics of model architectures tested in series 1004.

Training / validation: 75 / 25
No of hidden layers: 2 (*dim_hidden1* = 100 – 900, *dim_hidden2* = 50)
CV: 3-fold
Loss function: Categorical cross entropy

						None				Weakly active				Active			
**Run**	***dim_hidden1***	**Training accuracy**		**Validation accuracy**		**True positive**		**False positive**		**True positive**		**False positive**		**True positive**		**False positive**	
		**mean**	**std**	**mean**	**std**	**mean**	**std**	**mean**	**std**	**mean**	**std**	**mean**	**std**	**mean**	**std**	**mean**	**std**
1	100	0.97	0.00	0.90	0.00	0.99	0.00	0.01	0.00	0.00	0.00	1.00	0.00	0.55	0.00	0.45	0.00
2	200	0.96	0.00	0.90	0.00	0.99	0.00	0.01	0.00	0.02	0.00	0.98	0.00	0.58	0.00	0.42	0.00
3	300	0.98	0.00	0.90	0.00	0.99	0.00	0.01	0.00	0.02	0.00	0.98	0.00	0.68	0.00	0.32	0.00
4	400	0.99	0.00	0.89	0.00	0.98	0.00	0.02	0.00	0.01	0.00	0.99	0.00	0.61	0.00	0.39	0.00
5	500	0.99	0.00	0.90	0.00	0.99	0.00	0.01	0.00	0.01	0.00	0.99	0.00	0.61	0.00	0.39	0.00
6	600	0.99	0.00	0.89	0.00	0.98	0.00	0.02	0.00	0.02	0.00	0.98	0.00	0.71	0.00	0.29	0.00
7	700	0.99	0.00	0.89	0.00	0.98	0.00	0.02	0.00	0.02	0.00	0.98	0.00	0.65	0.00	0.35	0.00
8	800	0.99	0.00	0.89	0.00	0.98	0.00	0.02	0.00	0.03	0.00	0.97	0.00	0.68	0.00	0.32	0.00
9	900	0.99	0.00	0.89	0.00	0.99	0.00	0.01	0.00	0.01	0.00	0.99	0.00	0.71	0.00	0.29	0.00

Table S7. Classification metrics of model architectures tested in series 2001.

Training / validation: 75 / 25
No of hidden layers: 1 (*dim_hidden1* = 5 – 195)
CV: 3-fold
Loss function: Macro F1

						None				Weakly active				Active			
**Run**	***dim_hidden1***	**Training accuracy**		**Validation accuracy**		**True positive**		**False positive**		**True positive**		**False positive**		**True positive**		**False positive**	
		**mean**	**std**	**mean**	**std**	**mean**	**std**	**mean**	**std**	**mean**	**std**	**mean**	**std**	**mean**	**std**	**mean**	**std**
1	5	0.97	0.00	0.87	0.00	0.96	0.00	0.04	0.00	0.03	0.00	0.97	0.00	0.52	0.00	0.48	0.00
2	10	0.98	0.00	0.88	0.00	0.96	0.00	0.04	0.00	0.04	0.00	0.96	0.00	0.71	0.00	0.29	0.00
3	15	0.98	0.00	0.87	0.00	0.96	0.00	0.04	0.00	0.03	0.00	0.97	0.00	0.68	0.00	0.32	0.00
4	20	0.98	0.00	0.88	0.00	0.97	0.00	0.03	0.00	0.03	0.00	0.97	0.00	0.71	0.00	0.29	0.00
5	25	0.99	0.00	0.89	0.00	0.98	0.00	0.02	0.00	0.03	0.00	0.97	0.00	0.68	0.00	0.32	0.00
6	30	0.99	0.00	0.88	0.00	0.97	0.00	0.03	0.00	0.03	0.00	0.97	0.00	0.71	0.00	0.29	0.00
7	35	0.99	0.00	0.88	0.00	0.97	0.00	0.03	0.00	0.03	0.00	0.97	0.00	0.71	0.00	0.29	0.00
8	40	0.99	0.00	0.89	0.00	0.98	0.00	0.02	0.00	0.02	0.00	0.98	0.00	0.71	0.00	0.29	0.00
9	45	0.99	0.00	0.88	0.00	0.97	0.00	0.03	0.00	0.03	0.00	0.97	0.00	0.68	0.00	0.32	0.00
10	50	0.99	0.00	0.89	0.00	0.98	0.00	0.02	0.00	0.03	0.00	0.97	0.00	0.71	0.00	0.29	0.00
11	55	0.99	0.00	0.88	0.00	0.97	0.00	0.03	0.00	0.02	0.00	0.98	0.00	0.71	0.00	0.29	0.00
12	60	0.99	0.00	0.89	0.00	0.97	0.00	0.03	0.00	0.02	0.00	0.98	0.00	0.71	0.00	0.29	0.00
13	65	0.99	0.00	0.89	0.00	0.98	0.00	0.02	0.00	0.02	0.00	0.98	0.00	0.68	0.00	0.32	0.00
14	70	0.99	0.00	0.89	0.00	0.97	0.00	0.03	0.00	0.02	0.00	0.98	0.00	0.68	0.00	0.32	0.00
15	75	0.99	0.00	0.89	0.00	0.98	0.00	0.02	0.00	0.01	0.00	0.99	0.00	0.71	0.00	0.29	0.00
16	80	0.99	0.00	0.89	0.00	0.98	0.00	0.02	0.00	0.02	0.00	0.98	0.00	0.58	0.00	0.42	0.00
17	85	0.99	0.00	0.90	0.00	0.99	0.00	0.01	0.00	0.01	0.00	0.99	0.00	0.71	0.00	0.29	0.00
18	90	0.99	0.00	0.89	0.00	0.98	0.00	0.02	0.00	0.02	0.00	0.98	0.00	0.74	0.00	0.26	0.00
19	95	0.99	0.00	0.89	0.00	0.98	0.00	0.02	0.00	0.03	0.00	0.97	0.00	0.68	0.00	0.32	0.00
20	100	0.99	0.00	0.89	0.00	0.98	0.00	0.02	0.00	0.02	0.00	0.98	0.00	0.68	0.00	0.32	0.00
21	105	0.99	0.00	0.89	0.00	0.98	0.00	0.02	0.00	0.01	0.00	0.99	0.00	0.68	0.00	0.32	0.00

22	110	0.99	0.00	0.89	0.00	0.98	0.00	0.02	0.00	0.01	0.00	0.99	0.00	0.71	0.00	0.29	0.00
23	115	0.99	0.00	0.89	0.00	0.98	0.00	0.02	0.00	0.01	0.00	0.99	0.00	0.68	0.00	0.32	0.00
24	120	0.99	0.00	0.89	0.00	0.98	0.00	0.02	0.00	0.02	0.00	0.98	0.00	0.68	0.00	0.32	0.00
25	125	0.99	0.00	0.89	0.00	0.98	0.00	0.02	0.00	0.00	0.00	1.00	0.00	0.71	0.00	0.29	0.00
26	130	0.99	0.00	0.89	0.00	0.98	0.00	0.02	0.00	0.01	0.00	0.99	0.00	0.71	0.00	0.29	0.00
27	135	0.99	0.00	0.90	0.00	0.99	0.00	0.01	0.00	0.01	0.00	0.99	0.00	0.71	0.00	0.29	0.00
28	140	0.99	0.00	0.89	0.00	0.98	0.00	0.02	0.00	0.01	0.00	0.99	0.00	0.71	0.00	0.29	0.00
29	145	0.99	0.00	0.89	0.00	0.98	0.00	0.02	0.00	0.01	0.00	0.99	0.00	0.68	0.00	0.32	0.00
30	150	0.99	0.00	0.89	0.00	0.98	0.00	0.02	0.00	0.02	0.00	0.98	0.00	0.68	0.00	0.32	0.00
31	155	0.99	0.00	0.89	0.00	0.98	0.00	0.02	0.00	0.02	0.00	0.98	0.00	0.68	0.00	0.32	0.00
32	160	0.99	0.00	0.88	0.00	0.97	0.00	0.03	0.00	0.03	0.00	0.97	0.00	0.71	0.00	0.29	0.00
33	165	0.99	0.00	0.89	0.00	0.98	0.00	0.02	0.00	0.02	0.00	0.98	0.00	0.68	0.00	0.32	0.00
34	170	0.99	0.00	0.89	0.00	0.98	0.00	0.02	0.00	0.01	0.00	0.99	0.00	0.71	0.00	0.29	0.00
35	175	0.99	0.00	0.89	0.00	0.99	0.00	0.01	0.00	0.01	0.00	0.99	0.00	0.71	0.00	0.29	0.00
36	180	0.99	0.00	0.89	0.00	0.98	0.00	0.02	0.00	0.01	0.00	0.99	0.00	0.68	0.00	0.32	0.00
37	185	0.99	0.00	0.90	0.00	0.99	0.00	0.01	0.00	0.01	0.00	0.99	0.00	0.74	0.00	0.26	0.00
38	190	0.99	0.00	0.89	0.00	0.98	0.00	0.02	0.00	0.01	0.00	0.99	0.00	0.68	0.00	0.32	0.00
39	195	0.99	0.00	0.89	0.00	0.98	0.00	0.02	0.00	0.01	0.00	0.99	0.00	0.68	0.00	0.32	0.00

Table S8. **The 123 compounds used for the selection of prioritised set for experimental validation.**

Up to five compounds which are readily available via MolPort were randomly selected from each of 27 clusters formed within the 9,707 predicted "active" compounds as potential candidates for experimental testing. These 123 selected compounds passed the screening for potentially existing patent claims for anthelmintic activity by automated searches of the web resource Google Patents and evaluated by chemoinformatic means for drug-likeness and absence of known PAINs motifs. [#]The 10 compounds prioritised for the experimental evaluation are indicated next to the cluster number.

SMILES	Cluster	MolPort ID	MolPort availability (mg)	Number of patents	Adheres to Lipinski	PAINS
CC(C)(C)Sc1ccccc1N	0	MolPort-004-372-426	40000	3	TRUE	
CC(C)c1ccc([N+](=O)[O-])cc1	0	MolPort-001-768-103	200000	204	TRUE	
N#Cc1ccc(N)c(F)c1F	0	MolPort-001-778-123	50000	8	TRUE	
Cc1cc(F)c(F)c(C#N)c1	0	MolPort-002-462-207	4000	1	TRUE	
N#Cc1c(F)cc(F)cc1Br	0	MolPort-042-684-162	1000	2	TRUE	
CN(C)c1ccc(N=C(C#N)C#N)cc1	1	MolPort-004-922-629	50	0	TRUE	anil_di_alk_A(478)
CC(=O)c1ccc(C(C)(C)O)cc1	1	MolPort-028-950-569	2000	59	TRUE	
COc1c(C#N)c(C#N)c(OC)c(OC)c1OC	1	MolPort-001-905-836	10	0	TRUE	
C#CCNS(=O)(=O)c1c(C)cc(C)cc1C	1	MolPort-006-755-133	100	0	TRUE	
COc1cc(C#N)cc(NC=O)c1O	1	MolPort-035-881-153	300	0	TRUE	
COc1cc(NC(C)=S)ccc1SC	2	MolPort-002-318-652	300	1	TRUE	
COC(=O)c1ccc(SC)cc1	2	MolPort-001-765-958	12500	75	TRUE	
N#Cc1cccc(CO)c1F	2	MolPort-028-616-010	5000	1	TRUE	
O=C(O)COc1c(F)cccc1Br	2	MolPort-029-068-166	5000	0	TRUE	
O[C@H](CSc1ccccc1)C(F)(F)F	2	MolPort-000-006-557	5000	2	TRUE	
COC(=O)c1cnc(C)cc1C	3	MolPort-023-333-864	1000	2	TRUE	
CCCn1c(C)cc(C)c(C#N)c1=O	3	MolPort-008-753-266	1550	0	TRUE	
N#Cc1nc(O)c(O)nc1C#N	3	MolPort-000-657-288	20000	15	TRUE	
CSc1nc(N)c(C#N)c(CC(C)C)c1C#N	3	MolPort-003-179-995	1	0	TRUE	
N#Cc1cnc(C(=O)O)c(Cl)c1	3	MolPort-009-195-595	20000	32	TRUE	

N#Cc1cc(N)cc(Br)c1	4	MolPort-006-826-780	925000	17	TRUE
N#CCON	4	MolPort-027-631-770	25	13	TRUE
N#Cc1ccc(N)nc1Br	4	MolPort-047-976-293	2000	0	TRUE
N#CCCSCCO	4	MolPort-001-781-606	10000	11	TRUE
CNCCC#N	4	MolPort-001-791-512	500000	1537	TRUE
O=c1[nH]cnc2c1CCCC2	5	MolPort-003-894-437	1500	20	TRUE
Nc1ccc2c(c1)CCC(=O)N2	5	MolPort-000-000-609	69000	53	TRUE
N=C1CSc2ccccc2N1	5	MolPort-002-992-070	10000	10	TRUE
c1ccc(-c2cnc3ccccc3n2)cc1	5[#]	MolPort-001-014-341	1000	4546	TRUE
O=C1CCc2cc(Br)c(O)cc2N1	5	MolPort-027-720-039	25000	2	TRUE
Cc1cc(C)c(C#N)c(SC(C)(C)C)n1	6	MolPort-003-801-279	5000	0	TRUE
FC(F)(F)c1ccc(S)nc1	6	MolPort-000-145-614	525000	99	TRUE
FC(F)(F)c1cnc(Cl)nc1Cl	6	MolPort-000-003-502	4860000	1250	TRUE
N#CCc1cc(C(F)(F)F)ccn1	6	MolPort-020-916-095	5000	0	TRUE
Nc1c(F)cnc(F)c1F	6	MolPort-000-860-825	6000	10	TRUE
CC(C)(CO)NCC#Cc1ccccc1	7[#]	MolPort-000-863-118	50	0	TRUE
O=[N+]([O-])c1cccc(C#Cc2ccccc2)c1	7	MolPort-001-939-727	5000	6	TRUE
O=C(CCSc1ccccc1)NC1CC1	7	MolPort-002-086-934	50	0	TRUE
N#Cc1ccc(OCc2cccc(F)c2)cc1	7[#]	MolPort-002-290-750	100	5	TRUE
NC(=O)COc1ccccc1	7	MolPort-000-158-149	25000	19204	TRUE
C=CCNC(=S)/N=c1/ccccn1CC	8	MolPort-002-543-203	200	0	TRUE
O=C(CCC(F)(F)F)NCCc1cnccn1	8[#]	MolPort-035-859-252	10	0	TRUE
COC(=O)c1cncc(SC)c1	8	MolPort-028-748-771	1000	4	TRUE
N#CCCSc1ccc(Cl)cc1	8	MolPort-001-766-805	800	4	TRUE
CC(C)=CCOc1nccnc1C#N	8	MolPort-046-760-036	300	0	TRUE
O=S1(=O)Oc2ccccc2-c2ccccc21	9	MolPort-002-484-168	9000	0	TRUE
N#Cc1ccc(I)cn1	9	MolPort-006-717-160	10000	10	TRUE
N#Cc1cc(I)ccn1	9	MolPort-001-767-687	1000	17	TRUE
Cc1ccc(C=O)cc1I	9	MolPort-005-939-829	20000	23	TRUE
Cc1ccc2cc(N)ccc2n1	9	MolPort-000-141-701	50000	156	TRUE

Oc1ccc(/N=C/c2cccnc2)cc1	10	MolPort-001-023-713	30	0	TRUE	
COC(=O)c1ccc(-c2cc(C#N)ccn2)cc1	10#	MolPort-001-760-547	25000	0	TRUE	
Nc1cnc(NCC(F)(F)F)nc1	10	MolPort-012-218-632	1000	0	TRUE	
COc1ccc(-c2cncc(C#N)n2)cc1OC	10#	MolPort-003-803-914	10	0	TRUE	
Cc1cc(OC(F)(F)F)ccc1C(N)=O	10	MolPort-001-771-355	5000	9	TRUE	
CCC(CC)NC(=O)CSc1ccccc1	11	MolPort-001-550-548	100	0	TRUE	
CCCCOc1ccccc1CC(=O)O	11	MolPort-000-148-543	10	2	TRUE	
O=C(O)CCCCOCc1ccccc1	11	MolPort-005-247-610	2000	26	TRUE	
CCCCCOc1ccc(C=O)cc1OCC	11	MolPort-000-679-625	112000	4	TRUE	
CCCCCOC(=O)c1ccc(O)cc1	11	MolPort-003-719-476	1000000	403	TRUE	
N#Cc1ccc(C2(C(F)(F)F)N=N2)cc1	12	MolPort-046-933-109	3000	0	TRUE	
N#CCSc1nc2c(cc1C#N)CCCCC2	12	MolPort-001-968-446	2	0	TRUE	
CC1(C)CC(C#N)CC(C)(C)N1O	13	MolPort-004-963-831	1000	59	TRUE	
N#Cc1ccc(N2CCC(O)CC2)cc1	13#	MolPort-004-371-591	78250	13	TRUE	
N#CCN1CCC(N)CC1	13	MolPort-012-085-842	1000	5	TRUE	
CC1(C)COB(c2ccccc2C#N)OC1	13	MolPort-000-931-573	150000	15	TRUE	
N#CC1(N)CCCCC1	13	MolPort-002-466-106	175000	100	TRUE	
O=[N+]([O-])/C=C/c1ccccc1C(F)(F)F	14	MolPort-001-774-515	10800	19	TRUE	
N#Cc1ccc(C(F)(F)F)cc1	14	MolPort-000-158-998	28000000	836	TRUE	
OCc1cccc(C(F)(F)F)c1	14	MolPort-000-159-007	3300000	372	TRUE	
O=[N+]([O-])c1cc(F)c(F)cc1F	14	MolPort-000-159-190	7000000	292	TRUE	
Nc1ccc(F)c(OC(F)(F)F)c1O	14	MolPort-046-166-088	100	0	TRUE	
N#Cc1ccc(I)s1	15	MolPort-023-334-137	4000	5	TRUE	
Cc1ccc2[nH]c(C#N)cc2c1	15	MolPort-022-258-681	4000	8	TRUE	
N#Cc1cccc(-c2ccccc2)c1	15	MolPort-000-153-684	100000	493	TRUE	
N#Cc1csc(Br)c1	15	MolPort-022-264-647	2000	20	TRUE	
N#Cc1ccc(-c2cccc(F)c2)cc1	15	MolPort-000-928-561	5000	5	TRUE	
N#Cc1ccc(NC(=O)COCC(=O)NCC(N)=O)cc1	16#	MolPort-002-954-351	100	0	TRUE	
CCCCC(=O)c1ccc(OC)cc1	16	MolPort-001-789-177	25000	17	TRUE	
N#CC(C#N)/N=N\c1ccc([N+](=O)[O-])cc1	16	MolPort-000-814-964	200	0	TRUE	azo_A(324)

CC(C)(C)[S@](=O)/N=C/c1ccccc1	16	MolPort-039-062-280	10000	5	TRUE	
Cc1ccc(N/N=C(/C#N)C(N)=S)cc1	16	MolPort-002-931-819	400	0	TRUE	cyano_imine_A(37)
CCNC(=O)c1cccc(C#N)c1	17	MolPort-004-339-493	10000	6	TRUE	
N#CC(C#N)=NNc1ccc(F)cc1	17	MolPort-000-872-860	10000	1	TRUE	cyano_imine_B(17)
NC(=O)Cc1ccccc1O	17	MolPort-003-915-261	1000	42	TRUE	
N#Cc1ccc(C2CC2)nc1	17	MolPort-022-467-904	20000	7	TRUE	
CC(C)(CO)[C@H](N)c1ccccc1	17	MolPort-028-610-244	20000	0	TRUE	
COc1c2c(c(C)cc1C#N)CCC2	18	MolPort-000-141-871	100	0	TRUE	
N#Cc1sc2nc3c(cc2c1N)CCC3	18	MolPort-000-921-542	300	0	TRUE	
N#Cc1c(O)nc2c(c1C#N)CCCCCC2	18	MolPort-005-310-084	3	1	TRUE	
C#CC12CCC(CO)(CC1)CC2	19	MolPort-035-831-656	250	0	TRUE	
O=P(O)(O)CCc1ccccc1	20	MolPort-003-712-692	9000	567	TRUE	
O=[N+]([O-])c1cnc2c(c1)CCC2	20	MolPort-009-197-066	1000	2	TRUE	
C#CCSc1nc2c(cc1C#N)CCC2	20	MolPort-001-487-008	100	1	TRUE	
N#Cc1cc2c(c(F)c1)NCC2	20	MolPort-047-350-630	10000	0	TRUE	
CP(C)(=O)COc1ccccc1	20	MolPort-045-901-383	1	2	TRUE	
C=CCC#N	21	MolPort-001-779-791	100000	14436	TRUE	
C=CCN(C#N)CC=C	21	MolPort-000-005-431	600	333	TRUE	
C#CCOCCON	21	MolPort-046-024-011	46500	0	TRUE	
C#CCCCCC#N	21	MolPort-022-878-830	25000	15	TRUE	
CC(C)CN(CC#N)CC(C)C	21	MolPort-001-793-868	25000	5	TRUE	
Nc1ccc(S(=O)(=O)C(F)(F)F)cc1	22	MolPort-001-775-982	100000	82	TRUE	
N#Cc1cc(F)c(CN)c(F)c1	22	MolPort-038-542-860	250	5	TRUE	
CC(C)c1cc(C=O)cc(C(C)C)c1O	22	MolPort-002-144-464	5000	67	TRUE	
N#Cc1cc([N+](=O)[O-])c(F)cc1F	22	MolPort-001-772-729	1190000	32	TRUE	
Cc1cccc(OC(C)(C)C(=O)O)c1C	22	MolPort-000-889-587	10000	7	TRUE	
N#Cc1ccc(C2CNC2)cc1	23	MolPort-046-156-467	1250	15	TRUE	
N#Cc1cccc(C2OCCO2)c1	23	MolPort-039-138-501	60000	8	TRUE	
COc1ccc(Br)cc1/C=N/N	24	MolPort-035-924-048	20	0	TRUE	
C#CCOc1cccc(C=O)c1	24	MolPort-001-001-418	1000	21	TRUE	

N#CNCc1ccccc1Cl	24	MolPort-009-200-538	500	0	TRUE
OCCc1c(F)cccc1F	24	MolPort-004-782-222	20000	10	TRUE
O/N=C/c1ccc(Br)cc1F	24	MolPort-001-777-112	100	10	TRUE
N#CC1(N)CCSCC1	25	MolPort-000-001-264	7000	23	TRUE
OCC1=CNc2ccccc2N1	25	MolPort-039-008-733	100	0	TRUE
COC(=O)C1(C#N)C(C#N)(C(=O)OC)C1(C#N)C(=O)OC	25	MolPort-035-881-451	50	0	TRUE
N=C(N)c1ccc(N2CCOCC2)cc1	25[#]	MolPort-004-370-955	500	8	TRUE
CC12CN3CN(C1)CC(c1ccccc1)(C3)C2=O	25[#]	MolPort-001-974-153	300	0	TRUE
N#Cc1ncc(Cl)cc1O	26	MolPort-029-944-557	4000	4	TRUE
N#Cc1cncc(Cl)c1	26	MolPort-003-991-811	900000	40	TRUE
Cc1cccnc1C#N	26	MolPort-000-153-714	16000000	543	TRUE
Cc1cc(C=O)cc(C)n1	26	MolPort-003-824-375	17000	41	TRUE
Cc1cc(OC(F)F)ncc1Br	26	MolPort-008-146-616	13000	3	TRUE

Table S9. **Chemical identifiers of 10 small-molecule compounds predicted to be 'active' against *Haemonchus contortus* and two commercial anthelmintic compounds (monepantel and moxidectin) used as positive controls.**

Compound	SMILES	IUPAC name
**1**	COC(=O)c1ccc(-c2cc(C#N)ccn2)cc1	Methyl 4-(4-cyanopyridin-2-yl)benzoate
**2**	N=C(N)c1ccc(N2CCOCC2)cc1	4-(Morpholin-4-yl)benzene-1-carboximidamide
**3**	COc1ccc(-c2cncc(C#N)n2)cc1OC	6-(3,4-Dimethoxyphenyl)pyrazine-2-carbonitrile
**4**	c1ccc(-c2cnc3ccccc3n2)cc1	2-Phenylquinoxaline
**5**	O=C(CCC(F)(F)F)NCCc1cnccn1	4,4,4-Trifluoro-*N*-[2-(pyrazin-2-yl)ethyl]butanamide
**6**	N#Cc1ccc(OCc2cccc(F)c2)cc1	4-[(3-Fluorophenyl)methoxy]benzonitrile
**7**	N#Cc1ccc(N2CCC(O)CC2)cc1	4-(4-Hydroxypiperidin-1-yl)benzonitrile
**8**	CC12CN3CN(C1)CC(c1ccccc1)(C3)C2=O	(1S,5R,7S)-5-Methyl-7-phenyl-1,3-diazatricyclo[3.3.1.$1^{3,7}$]decan-6-one
**9**	N#Cc1ccc(NC(=O)COCC(=O)NCC(N)=O)cc1	*N*-(Carbamoylmethyl)-2-{[(4-cyanophenyl)carbamoyl]methoxy}acetamide
**10**	CC(C)(CO)NCC#Cc1ccccc1	2-Methyl-2-[(3-phenylprop-2-yn-1-yl)amino]propan-1-ol
Monepantel	CC(COC1=C(C=CC(=C1)C#N)C(F)(F)F)(C#N)NC(=O)C2=CC=C(C=C2)SC(F)(F)F	*N*-[(2*S*)-2-cyano-1-[5-cyano-2-(trifluoromethyl)phenoxy]propan-2-yl]-4-(trifluoromethylsulfanyl)benzamide
Moxidectin	CC1CC(=CCC2CC(CC3(O2)CC(=NOC)C(C(O3)C(=CC(C)C)C)C)OC(=O)C4C=C(C(C5C4(C(=CC=C1)CO5)O)O)C)C	(1*R*,4*S*,4'*E*,5'*S*,6*R*,6'*S*,8*R*,10*E*,13*R*,14*E*,16*E*,20*R*,21*R*,24*S*)-21,24-dihydroxy-4'-methoxyimino-5',11,13,22-tetramethyl-6'-[(*E*)-4-methylpent-2-en-2-yl]spiro[3,7,19-trioxatetracyclo[15.6.1.$1^{4,8}$.$0^{20,24}$]pentacosa-10,14,16,22-tetraene-6,2'-oxane]-2-one

Table S10. Chemical identifiers of 20 small-molecule compounds randomly selected from the ZINC15 database.

These compounds were predicted to be inactive ('none') against *Haemonchus contortus* and were utilised as negative controls (non-actives) in the validation assay.

Compound	SMILES	IUPAC name
**11**	C1(C(C(O)C(OC1OC)CO)O)O	2-(hydroxymethyl)-6-methoxytetrahydro-2H-pyran-3,4,5-triol
**12**	C(=O)(OC)CNCC(=O)OC.Cl	methyl 2-[(2-methoxy-2-oxoethyl)amino]acetate hydrochloride
**13**	N1C(C(=O)NCC1=O)C(C)C	3-isopropylpiperazine-2,5-dione
**14**	N1=C(NCN(C1)CCCO)SC.Br	3-[4-(methylthio)-1,2,3,6-tetrahydro-1,3,5-triazin-1-yl]propan-1-ol hydrobromide
**15**	S1(CC2C(S2)C1)(=O)=O	tetrahydro-1aH-3lambda~6~-thiireno[2,3-c]thiophene-3,3-dione
**16**	C1(=C(C(=O)C=C(O1)CO)O)CO	3-hydroxy-2,6-di(hydroxymethyl)-4H-pyran-4-one
**17**	C(CCSCCC(=O)N)(=O)N	3-[(3-amino-3-oxopropyl)thio]propanamide
**18**	N1C(NC(C1=O)CCSC)=O	5-[2-(methylthio)ethyl]imidazolidine-2,4-dione
**19**	N12C(NC3=C(C1=O)CCC3)=NC=N2	4,5,6,7-tetrahydro-8H-cyclopenta[d][1,2,4]triazolo[1,5-a]pyrimidin-8-one
**20**	O1C(C(O)C(C1C(O)CO)O)=O	5-(1,2-dihydroxyethyl)-3,4-dihydroxytetrahydrofuran-2-one
**21**	C=1(C(NC(NC1)=O)=O)N2CCOCC2	5-morpholino-2,4(1H,3H)-pyrimidinedione
**22**	C(C(O)CO)(C(O)CO)O	pentane-1,2,3,4,5-pentol
**23**	N1C(C(CCC(=O)O)=NNC1=O)=O	3-(3,5-dioxo-2,3,4,5-tetrahydro-1,2,4-triazin-6-yl)propanoic acid
**24**	C1(NCN(CN1)CCO)=S	5-(2-hydroxyethyl)-1,3,5-triazinane-2-thione
**25**	C(=N)(NCCCC(C(=O)O)N)N.Cl	L-arginine hydrochloride
**26**	N1(C(N(C)C(N(C1=O)C)=O)=O)C	1,3,5-trimethyl-1,3,5-triazinane-2,4,6-trione
**27**	N1(C(C(=O)O)CC(C1)O)C(=O)C	1-acetyl-4-hydroxypyrrolidine-2-carboxylic acid
**28**	C1(=NN(C)C(=C1)O)C(=O)OC	methyl 5-hydroxy-1-methyl-1H-pyrazole-3-carboxylate
**29**	N12C(NC(C1CSC2)=O)=O	perhydroimidazo[1,5-c][1,3]thiazole-5,7-dione
**30**	C1(=C(OCC)C(C1=O)=O)NN(C)C	3-(2,2-dimethylhydrazino)-4-ethoxycyclobut-3-ene-1,2-dione

Table S11. **Similarity of compounds 3 and 6 with compounds that possess antiparasitic activity.**

The APDB identifier refers to the AntiparasiticsDB (https://antiparasiticsdb.org) where further details regarding bioactivity and links to the original literature reports can be found.

Compound **3**: COC1=C(C=C(C=C1)C2=NC(=CN=C2)C#N)OC

Identifier	SMILES	Tanimoto similarity	Bioactivity against
APDB C798	C3C1=C(N=CC(=N1)C2=CC(=C(C=C2)OC)C#N)CCC3	0.45	*Haemonchus contortus*
APDB C799	C1CCCC2=C1N=C(C=N2)C3=CC(=C(C=C3)OC)Cl	0.50	*Haemonchus contortus*
APDB C802	C1CCCC2=C1N=C(C=N2)C3=CC=CC(=C3)OC(C)C	0.61	*Haemonchus contortus*

Compound **6**: C1=CC(=CC(=C1)F)COC2=CC=C(C=C2)C#N

Identifier	SMILES	Tanimoto similarity	Bioactivity against
ABDP C823, PubChem CID 11224875, CHEMBL402864	CC(COC1=C(C=CC(=C1)C#N)C(F)(F)F)(C#N)NC(=O)C2=CC=C(C=C2)OC(F)(F)F	0.21	*Dirofilaria immitis, Haemonchus contortus, Trichostrongylus colubriformis*
APDB C836	C1=C[CH]C=C1.C2=C([CH]C=C2)COC3=C(C=C(C=C3)C#N)C(F)(F)F.[Fe]	0.27	*Dirofilaria immitis, Haemonchus contortus, Trichostrongylus colubriformis*

Table S12. Compound library assembled as a subset of the ZINC15 database and used in the *in silico* screening.

**Tranche MW category**	**Tranche LogP category**	**MW (Da)**	**Log*P***
A	A – G	MW ≤ 200	-1 < Log*P* ≤ 3.5
B	A – G	200 < MW ≤ 250	-1 < Log*P* ≤ 3.5
C	A – G	250 < MW ≤ 325	-1 < Log*P* ≤ 3.5
D	A – G	325 < MW ≤ 350	-1 < Log*P* ≤ 3.5
E	A – G	350 < MW ≤ 375	-1 < Log*P* ≤ 3.5
F	A – G	375 < MW ≤ 400	-1 < Log*P* ≤ 3.5
G	A – F	400 < MW ≤ 425	-1 < Log*P* ≤ 3.0
H	A – F	425 < MW ≤ 450	-1 < Log*P* ≤ 3.0
I	A – F	450 < MW ≤ 475	-1 < Log*P* ≤ 3.0
J	A – F	475 < MW ≤ 500	-1 < Log*P* ≤ 3.0
K	A – F	MW > 500	-1 < Log*P* ≤ 3.0

Figure S1. The dose-response assessment of *in vitro* motility inhibition of 10 prioritised small molecules from ZINC database, predicted to be 'active' against *Haemonchus contortus*.

Compounds (see S2 Table) were assessed using exsheathed third-stage larvae (xL3s) of *H. contortus*, with reference to two control compounds, monepantel (MON) and moxidectin, (MOX). Dose-response curves show the inhibition of *H. contortus* motility was observed for all 10 compounds at 168 h.

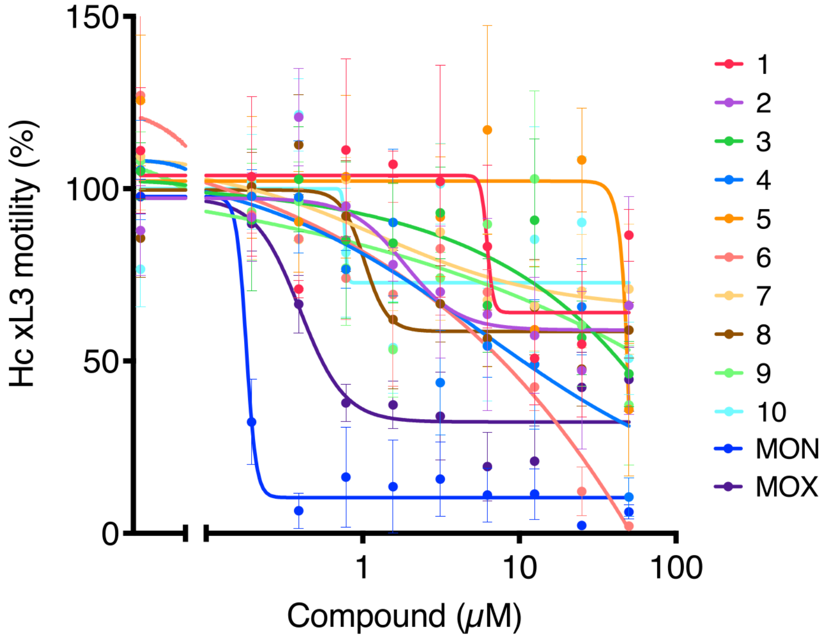

Figure S2. The dose-response assessment of *in vitro* motility inhibition of 20 randomly chosen small molecules from ZINC database, predicted to be inactive ('none') against *Haemonchus contortus*.

Compounds (see S3 Table) were assessed using exsheathed third-stage larvae (xL3s) of *H. contortus*, with reference to two 'active' compounds (**3** and **6**). Dose-response curves show the inhibition of *H. contortus* motility was only observed for compounds **3** and **6** at 168 h.

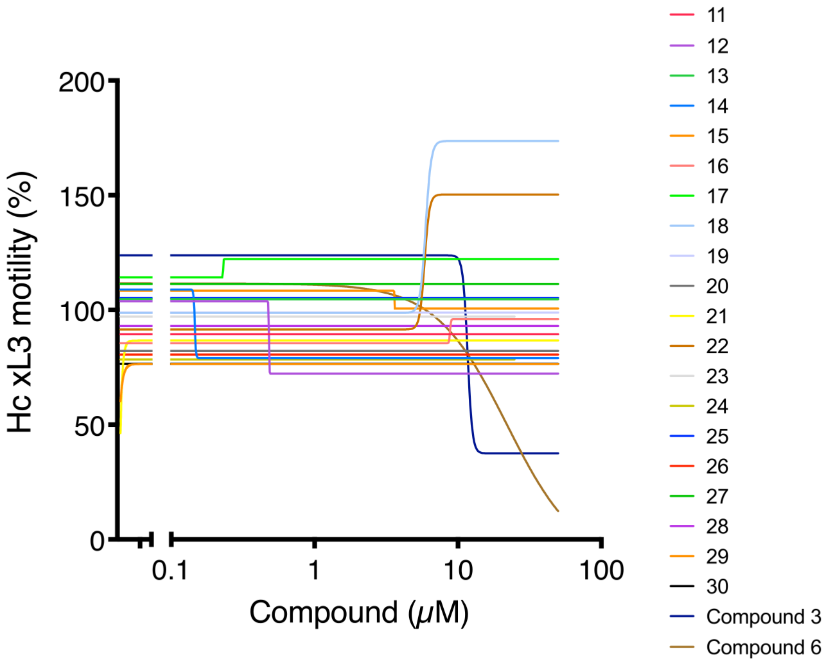

Supplement: Supplementary file 1 [file ijms-26-03134-s001.zip › ijms-3480558-supplementary.pdf]
